# Chrysogenones A–E: Malonyl-Modified Ergosterone Derivatives from Deep-Sea-Derived *Penicillium* sp. MCCC 3A00121 as Inhibitors of Renal Fibroblast Activation

**DOI:** 10.3390/md24030121

**Published:** 2026-03-23

**Authors:** Zeqing Li, Lei Chen, Yuan Wang, Mengjiao Jiang, Siyu Fang, Rong Chao, Taizong Wu, Tianhua Zhong

**Affiliations:** 1 Key Laboratory of Marine Genetic Resources, Third Institute of Oceanography, Ministry of Natural Sources, 184 Daxue Road, Xiamen 361005, China; rankqing@163.com (Z.L.); wangyuan@tio.org.cn (Y.W.); 18979858680@163.com (S.F.); chaorong@tio.org.cn (R.C.); 2 Shenzhen Key Laboratory of Chinese Medicine Active Substance Screening and Translational Research, Department of Pharmacy, The Seventh Affiliated Hospital, Sun Yat-Sen University, Shenzhen 518107, China; chenl355@alumni.sysu.edu.cn (L.C.); jiangmj33@mail2.sysu.edu.cn (M.J.); 3 Key Laboratory of Microbial Pathogenesis and Interventions of Fujian Province University, The Key Laboratory of Inmate Immune Biology of Fujian Province, Biomedical Research Center of South China, College of Life Sciences, Fujian Normal University, Fuzhou 350117, China

**Keywords:** deep-sea, *Penicillium*, steroids, renal fibrosis, network pharmacology, molecular docking

## Abstract

Five previously undescribed steroids, chrysogenones A–E (**1**–**5**), were isolated from the deep-sea-derived *Penicillium* sp. MCCC 3A00121. Their chemical structures were unambiguously established through comprehensive spectroscopic analyses, density functional theory (DFT)-based electronic circular dichroism (ECD) calculations, and X-ray crystallography. Chrysogenones represent a class of oxidatively modified ergosterone-type derivatives, with **1**, **2**, and **5** featuring an uncommon malonyl substitution at C-12 of the ergosterone skeleton. Biologically, **1**–**5** exhibited varying degrees of inhibitory activity against renal fibrosis, as evidenced by the downregulation of the key fibrotic markers α-smooth muscle actin (α-SMA) and collagen I (COL1A1). Among them, chrysogenone B (**2**) emerged as the most promising candidate, demonstrating superior potency and pronounced inhibition of activated NRK-49F cell proliferation. Integrated network pharmacology analysis and molecular docking studies further suggested that the anti-renal fibrotic effects of compound **2** may be mediated through its interaction with putative molecular targets, including AKT1, HSP90AA1, and MDM2.

## 1. Introduction

Marine organisms have long been recognized as valuable sources for the discovery of bioactive natural products (NPs), owing to the extreme environmental conditions—such as perpetual darkness, low temperatures, hypoxia, and hyper salinity—that distinguish their habitats from terrestrial ecosystems [1,2,3]. To date, approximately 40,000 marine-derived NPs have been documented, with over 20 approved as marketed drugs and a growing number in clinical trials [4,5,6]. Although many of these pharmaceuticals, including cytarabine and eribulin from sponges, ziconotide from cone snails, and the oligosaccharide GV971 from brown algae, originate from marine invertebrates or macroalgae, marine fungi have emerged as a prolific source of novel NPs over the past decade [5,7,8,9]. Steroids represent one of the most significant classes of fungal metabolites, fulfilling essential biological roles such as serving as structural components of fungal cell membranes (e.g., ergosterol) and acting as signaling molecules [10,11]. Recently, ergosterol-derived steroids possessing immunosuppressive, antiproliferative [12,13], anti-inflammatory [14], antimicrobial activities [15] have been discovered from marine fungi, exhibiting a considerable potential of this compound class as drug-like candidates.

Renal fibrosis represents the common pathological endpoint of chronic kidney disease (CKD), characterized by the excessive accumulation and deposition of extracellular matrix (ECM) proteins, predominantly collagens, within the kidney parenchyma [16,17]. The pathogenesis is driven by persistent inflammation, chronic hypoxia, and dysregulated cellular signaling. Key mechanisms involve the activation of resident fibroblasts into matrix-producing myofibroblasts, epithelial–mesenchymal transition (EMT), and the sustained release of pro-fibrotic cytokines, most notably transforming growth factor-beta (TGF-β) [18]. Currently, no therapies specifically target renal fibrosis in clinical practice, which makes it a critical need for the discovery of designated pharmacological agents.

**Figure 1:**
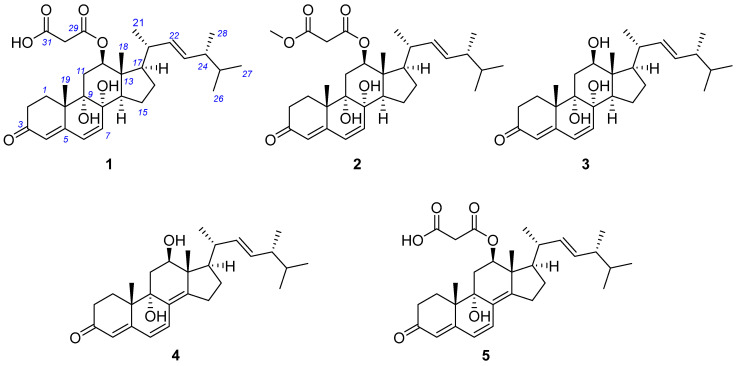
Structures of compounds **1**–**5** from *Penicillium* sp. MCCC 3A00121.

As part of our ongoing search for bioactive and structurally new compounds from marine fungi [19,20], we undertook a chemical investigation into the deep-sea-derived *Penicillium* sp. MCCC 3A00121. This led to the discovery of five previously undescribed steroids, chrysogenones A–E (**1**–**5**), which feature an oxidatively modified ergosterone scaffold (Figure 1). Compounds **1**–**5** exhibited significant inhibitory activity against renal fibrosis. Herein, we report the isolation, structural elucidation, and anti-fibrotic activities of these new metabolites.

## 2. Results

### 2.1. Structure Elucidation

The EtOAc extract obtained from the corn medium cultivation of *Penicillium* sp. MCCC 3A00121 was subjected to solvent trituration, followed by consecutive purifications through silica gel column chromatography and reversed-phase fractionation to yield **1**–**5**.

**Figure 2:**
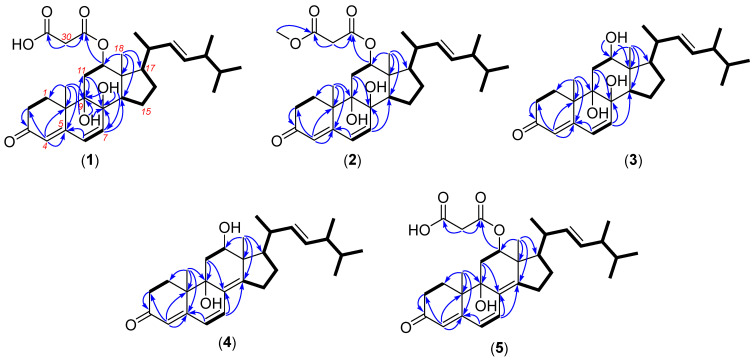
Selected COSY correlations (bold line) and HMBC correlations (blue arrows) of **1**–**5**.

Compound **1** was obtained as a white powder. Its chemical formula was determined to be C_31_H_44_O_7_ (Δmmu +0.5) based on its protonated peaks at *m/z* 551.2950 ([M + Na]^+^) in the HRESIMS spectrum, requiring ten degrees of unsaturation. Analysis of the ^1^H and ^13^C NMR spectra (Table 1) revealed the resonances for six methyls, including singlets and four doublets, six sp^3^ methylenes, eleven methines, including five olefinic and six sp^3^ methines, three carbonyl carbons, as well as six nonhydrogenated carbons, including one olefinic one, accounting for six degrees of unsaturation. Therefore, the molecule incorporates four rings. The ^1^H-^1^H COSY correlations of H_2_-1/H_2_-2, H-6/H-7, H-12/H_2_-11, H-20/H_3_-21, H-24/H_3_-28, H-25/H_3_-27, and H_2_-15/H_2_-16/H-17, along with the HMBC cross-peaks observed from H_3_-19 to C-1/5/9/10, H_3_-18 to C-12/13/14/17, H_2_-11 to C-8/9, H-7 to C-8/14, H-4 to C-2/5/10, H_2_-2 to C-3, as well as OH-8 to C-7/8/9, and OH-9 to C-8/9/11, permitted the construction of a tetracyclic, 3-ketone-8,9-dihydroxy-steroid framework (Figure 2). The COSY correlations of H-17/H-20/H-22/H-23/H-24/H-25/H_3_-26 indicated the side chain of the steroid positioned at C-17. The vinyl protons at *δ*_H_ 5.29 (1H, dd, *J* = 15.3, 8.4 Hz) and 5.24 (1H, dd, *J* = 15.3, 7.4 Hz) were characteristic signals for the *trans* Δ^22^ double bond found commonly in the side chains of some steroids. A further substitution at the oxygenated C-12 was assigned a malonyl group that was evident from the HMBC cross-peak from H-12 to C-29 and from the deshielding methylene CH_2_-30 (*δ*_H_ 3.32; *δ*_C_ 42.1) to the carbonyl C-29 and C-31. Therefore, the planar structure of **1** was assigned as shown (Figure 2).

**Figure 3:**
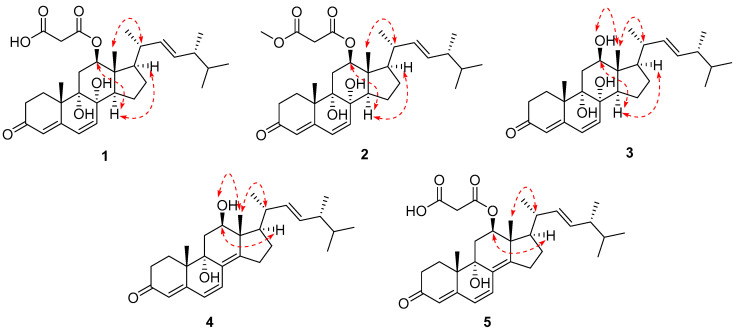
Selected NOESY correlations (red dotted line) of **1**–**5**.

**Figure 4:**
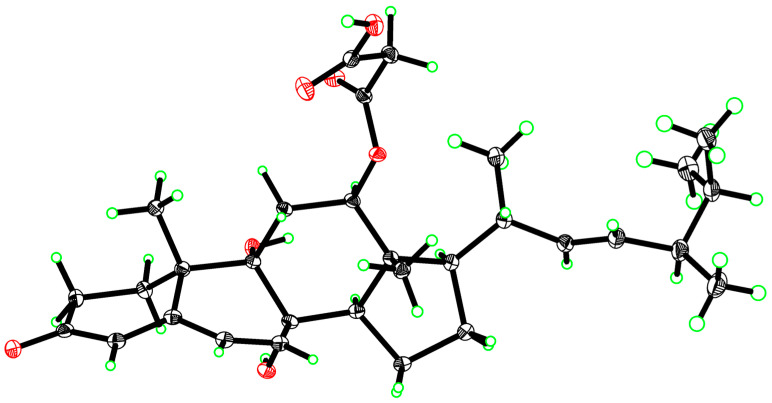
Single-crystal X-ray crystallography of **1**.

In the NOESY spectrum, diagnostic correlations observed between H-12 and H-14, H-14 and H-17, H_3_-18/H-20 (in DMSO) suggested that those protons were on the same side, thus determining the relative configuration among C-12/13/14/17 (Figure 3). However, the configurations of the remaining chiral centers are problematic due to either a lack of NOESY signals or the flexible nature of the acyclic side chain. Fortunately, the single crystal of compound **1** was obtained in methanol by slowly evaporating, with the absolute configuration of **1** unambiguously established by an X-ray diffraction analysis (Figure 4). Therefore, the complete structure of **1** was drawn as shown and given the trivial name chrysogenone A.

**Figure 5:**
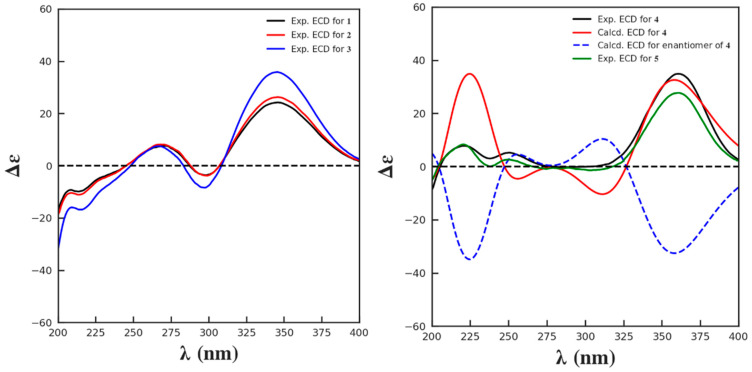
Calculated and experimental ECD spectra of **1**–**5**.

HRESI(+)MS measurement of compound **2** revealed the molecular formula C_32_H_46_O_7_ (Δmmu +2.0), requiring ten degrees of unsaturation, suggestive of a homolog of **1**. Comparison of the NMR spectra of **2** and **1** (Table 1) highlighted extreme similarity, except that **2** bears one more oxymethyl (*δ*_H_ 3.62; *δ*_C_ 52.1) positioned at the carbonyl C-31, evident by the HMBC correlation from the oxymethyl to C-31. Due to the biogenetic relationship between **2** and **1**, as well as the considerable agreement of their experimental CD curves (Figure 5), the absolute configuration of **2** was assumed to be consistent with that of **1**. Therefore, the complete structure of **2** was established and named chrysogenone B.

The molecular formula of compound **3** was determined to be C_28_H_42_O_4_ (Δmmu +5.3), as indicated by its HRESIMS spectrum, indicating eight degrees of unsaturation. Comparison of the NMR data of **3** with those of **1** and **3** (Table 1) revealed a high degree of similarity, with the primary difference being the shielding of H-12 (δ_ΔH_ = −1.40–1.42 ppm) as well as the replacement of the malonyl group with hydroxy, supported by the COSY correlation of H-12/OH-12 and HMBC cross-peak from OH-12 to C-12/13. Similar NOESY correlations of H-12/H-14, H14/H-17, and H_3_-18/H-20 were observed in **3**. This, along with a biogenetic consideration and the consistency of experimental CD spectra of **3** with those of **1**–**3** (Figure 5), permitted the same absolute configuration of **3** as **1**–**3**. Therefore, the structure of **3** was assigned and named chrysogenone C.

HRESI(+)MS analysis of compound **4** returned the molecular formula C_28_H_40_O_3_ (Δmmu −3.2), requiring nine degrees of unsaturation. The NMR spectra of **4** resembled those of **3**, with the primary difference being the replacement of two *sp^3^* carbons with two olefinic carbons, which accounts for the additional degree of unsaturation in **4** compared to **3**. A scrutiny of the HMBC spectrum of **4** established the double bond formed between C-8 and C-14 by the correlations from H_3_-18/H-7 to C-14, and H-7/H_2_-11 to C-8, while the remaining planar structure remains the same as **3** (Figure 2). In the NOESY spectrum, correlations of H-12/H-17 and H_3_-18/H-20 indicated that those protons were on the same side, consistent with the relative configurations among C-12/13/14 in **1**–**3**. Due to a conserved biosynthesis of the steroid framework in **1**–**3**, the absolute configuration of **4** was assumed to be identical to that of **1**–**3**. This assignment was further supported by ECD calculation for the predicted isomer (**6S**,**8S**,**9S**,**12R**,**13R**,**14R**,**17R**,**20R**,**24R**)-**1**, whose theoretical ECD curve exhibited good fitness with that of experimental data (Figure 5). Therefore, **4** was assigned the structure as shown and named chrysogenone D.

The molecular formula of compound **5** was identified as C_31_H_42_O_6_ (Δmmu −5.6) based on its protonated HRESI(+)MS peak at *m*/*z* 511.3004, suggestive of eleven degrees of unsaturation. Comparison of the NMR data of **5** (Table 2) with those of **4** revealed a high degree of similarity, with the primary difference being the deshielding of H-12 (δ_ΔH_ = +1.43 ppm) as well as the presence of resonances for a malonyl group instead of a hydroxy positioned at C-12. This assignment was well supported by the HMBC cross-peak from H-12 to C-29 and from the deshielding methylene CH_2_-30 (*δ*_H_ 3.39; *δ*_C_ 42.1) to the carbonyl C-29 and C-31. Likewise, the similar NOESY correlations of H-12/H-14, H14/H-17, and H_3_-18/H-20 observed in **5**, together with a biogenetic consideration and the consistency of experimental CD spectra of **5** with those of **4,** permitted the identical absolute configuration of **5** as **4** (Figure 5). Therefore, the structure of **5** was assigned and named chrysogenone E.

Malonyl substitution is relatively uncommon in natural products, despite malonyl-CoA serving as a key biosynthetic precursor for polyketides. To date, this structural unit has been reported in peptides [21], triterpenes [22,23,24], diterpenes [25], originating from sources such as plants, fungi, and pathogenic bacteria. Chrysogenones A–B and D (**1**–**2**, **5**) feature a malonyl group at the C-12 position of the steroid skeleton, representing rare examples within this class of natural products. Furthermore, the steroid core of compounds **1**–**5** displays distinct oxidation patterns at C-9, 12, and/or C-8 compared to their structurally related analogs 9*R*,11*R*dihydroxyergosta-4,6,8(14),22-tetraen-3-one [26], and isocyathisterol [27].

### 2.2. Anti-Renal Fibrosis Activity

**Figure 6:**
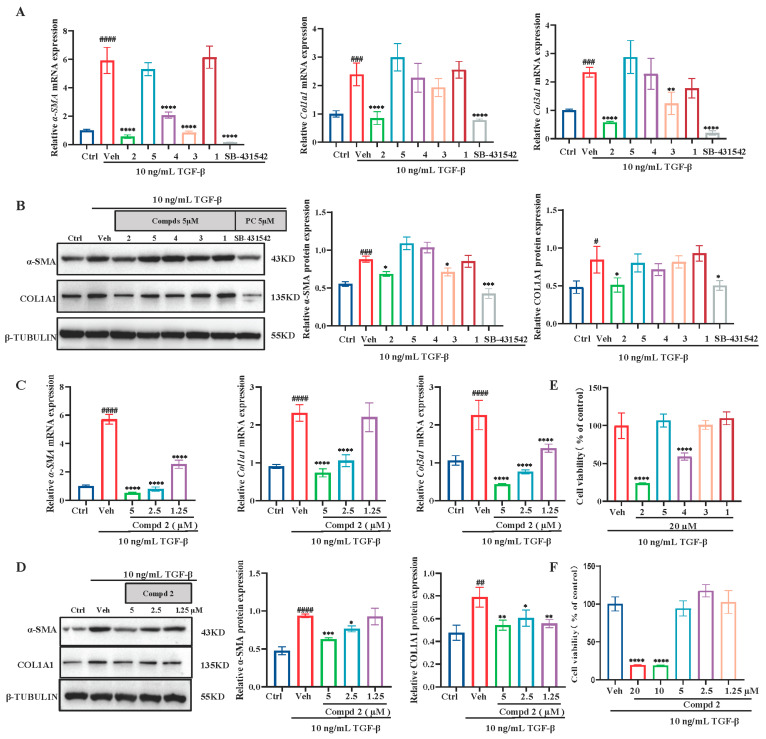
Identification of compound **2** as an anti-fibrotic compound in TGF-β1-activated renal fibroblasts. (**A**,**B**): mRNA and protein expression of fibrotic markers were assessed via quantitative real-time PCR (**A**) and Western blot (**B**) following 48 h treatment with vehicle or 5 μM of compounds **1**–**5**. (**C**,**D**): Dose-dependent effects of compound **2** on the mRNA expression of *α-Sma, Col1a1*, and *Col3a1* (**C**) and the protein expression of α-SMA and COL1A1 (**D**) were measured after 48 h of treatment at the indicated concentrations. (**E**,**F**): Cytotoxicity was evaluated using a CCK-8 assay after 24 h. Cell viability was assessed following treatment with 20 μM of compounds **1**–**5** (**E**) and with indicated concentrations of compound **2** (**F**). In all panels, NRK-49F cells were activated with 10 ng/mL TGF-β1. SB431542, a TGF-β receptor kinase inhibitor, served as a positive control where indicated. Data are presented as mean ± SEM and were analyzed using one-way ANOVA with Bonferroni’s multiple comparisons test. Significance is denoted as follows: # *p* < 0.05, ## *p* < 0.01, ### *p* < 0.001, #### *p* < 0.0001 vs. ctrl group; * *p* < 0.05, ** *p* < 0.01, *** *p* < 0.001, **** *p* < 0.0001 vs. vehicle group.

Given the established role of activated renal fibroblasts as drivers of kidney fibrosis, we investigated the effects of chrysogenones A–E (**1**–**5**) using an in vitro model of TGF-β1-stimulated rat kidney fibroblasts (NRK-49F) [28]. Treatment with TGF-β1 (10 ng mL^−1^, 48 h) robustly induced mRNA and protein expression of key fibrotic markers, confirming a stable pro-fibrotic phenotype (Figure 6A,B). Screening of the compounds revealed that, despite sharing a common structural scaffold, they exhibited distinct inhibitory profiles in attenuating TGF-β1-induced pro-fibrotic response at a concentration of 5 µM (Figure 6A,B). Among them, compound **2** demonstrated the strongest activity, significantly downregulating mRNA and protein levels of α-smooth muscle actin (α-SMA) and collagen I (COL1A1) compared to the TGF-β1 group (Figure 6A,B). The anti-renal fibroblast activation effect of compound **2** was confirmed to be dose-dependent across multiple markers at non-cytotoxic concentrations (Figure 6C,D).

In addition to renal fibroblast activation, the proliferation of activated fibroblasts contributes to fibrotic lesion expansion [29]. We therefore examined the effect of chrysogenones A–E on fibroblast proliferation. As a result, chrysogenone B (**2**) and chrysogenone D (**4**) showed pronounced inhibition of activated NRK-49F cell proliferation at concentrations above 20 µM, with **2** exhibiting the greatest potency (Figure 6E,F). By contrast, none of those compounds exhibited cytotoxicity on normal NRK-49F cells at above concentration (Figure S36). Taken together, these results identify chrysogenone B (**2**) as the most effective congener in inhibiting both fibrotic marker expression and fibroblast proliferation, warranting its selection for further mechanistic study.

### 2.3. Anti-Renal Fibrosis Target Prediction for Chrysogenone B (**2**)

**Figure 7:**
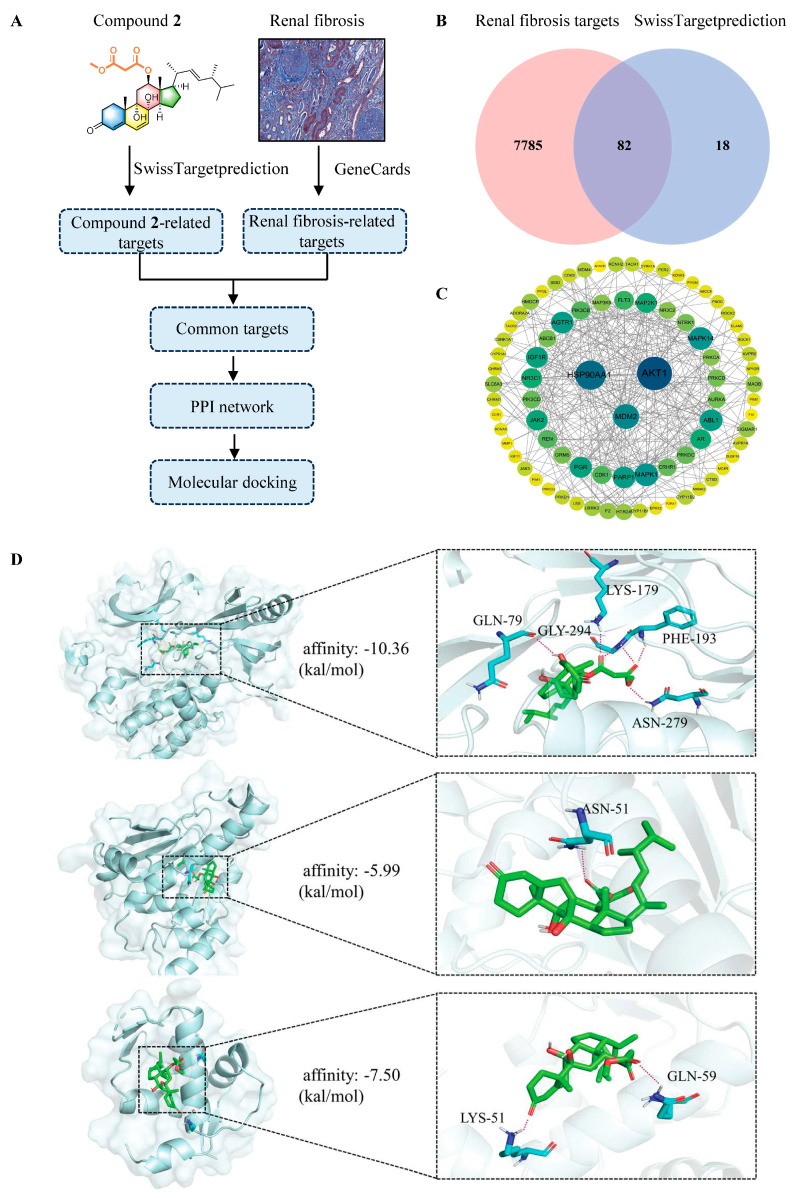
Anti-renal fibrosis target prediction for chrysogenone B (**2**). (**A**): Overall workflow of prediction of molecular target of **2**. (**B**): Overlaps between compound **2**-related targets and renal fibrosis-related targets. (**C**): PPI of overlapped targets. (**D**): Binding modes between compound **2** and the three candidate targets, AKT1 (top), HSP90AA1 (middle), MDM2 (bottom), with hydrogen bonds drawn in dotted lines.

To investigate the potential molecular target of compound **2** underlying its renal fibrosis inhibitory activity, we performed a theoretical analysis integrating network pharmacology and molecular docking. The overall workflow is summarized in Figure 7A. A representative histology image of renal fibrosis was sourced from the Kidney Tissue Atlas database [https://atlas.kpmp.org/ (accessed on 20 January 2026)]. A total of 100 compound **2**-related targets were obtained from the SwissTargetPrediction database, while 7867 renal fibrosis-related targets were collated from the GeneCards database. Intersection of these datasets using the Venny platform revealed 82 overlapping genes, constituting putative targets for the compound **2** in renal fibrosis (Figure 7B). To prioritize core targets from this set, we constructed a protein–protein interaction (PPI) network for the 82 candidates using the STRING database and visualized it in Cytoscape (v3.9). Network topology was analyzed with the CytoHubba plugin. Based on the Degree algorithm, three genes—AKT1, HSP90AA1, and MDM2—were identified as top-ranked hubs, representing key candidate targets for therapeutic intervention (Figure 7C). In this regard, these three core targets of compound **2** for renal fibrosis were chosen for further molecular docking.

Molecular docking studies indicate that compound **2** binds strongly to the three putative target proteins: AKT1 (PDB: 6HHG; Vina score: −10.36 kcal/mol), HSP90AA1 (PDB: 3BM9; Vina score: −5.99 kcal/mol), and MDM2 (PDB: 4OBA; Vina score: −7.5 kcal/mol) (Figure 7D). Notably, the binding pose of compound **2** within AKT1 closely resembles that of known AKT1 inhibitors, positioning it at the interface between the kinase and pleckstrin homology (PH) domains [30]. This interaction is stabilized by six hydrogen bonds with the key residues GLN-79, LYS-179, GLY-294, PHE-193, and ASN-279, most of which are contributed by the malonyl substitution. This likely explains the superior activity of compound **2** compared to compounds **3** and **4**. Despite the high structural similarity within compounds **1**, **2**, and **5**, we hypothesized that the observed activity variation might be associated with differences in their lipophilic properties (**2**: malonyl methyl ester; **1** and **5**: malonic acid), which could impact cell penetration. However, such findings are preliminary and require further experimental support in the future.

## 3. Materials and Methods

### 3.1. General Experimental Procedures

NMR spectra were recorded on a Jeol 600 MHz (JEOL Ltd., Tokyo, Japan) and a Bruker 400 MHz. ECD (Nantucket, MA, USA) spectra were measured on a Chirascan spectrometer (Applied Photophysics, Surrey, UK). Optical rotations were measured with an Anton Paar MCP100 polarimeter (Graz, Austria). The HRESIMS spectra were measured on a Waters Xevo G2 Q-TOF mass spectrometer (Waters Corporation, Milford, MA, USA). The semi-preparative HPLC was conducted on an Agilent Technologies 1260 Infinity instrument (Shanghai Yuanxi Instrument Co., Ltd., Shanghai, China) equipped with a DAD detector. The reversed-phase C-18 medium-pressure liquid chromatography (MPLC) was soochow high tech chromatography. Column chromatography (CC) was performed on silica gel (100–200 m, 200–300 m, 300–400 m; Qiaodao Marine Chemistry Co., Ltd., Qingdao, China), Sephadex LH-20 (Amersham Pharmacia Biotech AB, Uppsala, Sweden), and ODS (octadecylsilyl; 50 μm, Daiso, Hiroshima, Japan), respectively. The NRK-49F cells (rat renal fibroblast cells) were purchased from Wuhan Purcell Life Science and Technology Co., Ltd., Wuhan, China.

### 3.2. Fungal Strain and Identification

The fungal strain MCCC 3A00121 was isolated from a deep-sea sediment sample of the western Pacific Ocean at a depth of 2682 m. It was identified to be *Penicillium* sp. or *Penicillium* cf *chrysogenum,* as the ITS gene sequence alignment (PQ623891) demonstrated that it showed great similarity (99.8%) to *Penicillium chrysogenum* (GenBank accession number NR_077145.1). The strain was preserved at the Key Laboratory of Marine Genetic Resources, Third Institute of Oceanography, Ministry of Natural Resources (Xiamen, China).

### 3.3. Fermentation, Extraction, and Isolation

The fermentation was reconducted in 100 Erlenmeyer flasks (1000 mL), each containing 80 g of corn and 100 mL of artificial seawater, and the contents were soaked for 1 h before autoclaving for 20 min at 15 psi. Each flask was inoculated with 5.0 mL of the spore inoculum and incubated at r. t. for 30 days. The fermented materials were extracted with EtOAc (3 × 1000 mL) in an ultrasonic bath at 25 °C for 30 min to obtain brown crude extract (51.6 g). The crude extract (36.5 g) was separated into eight fractions (Fr.1–Fr.8) via CC over silica gel using a gradient of EtOAc-PE (0% → 100%, 8 h, 20 mL/min). Fraction Fr,4 (3.4 g) was subjected to column chromatography on ODS silica gel eluted with MeOH/H_2_O to give five subfractions (Fr.4.1–Fr.4.5). Fr.4.3 was separated by HPLC using MeOH/H_2_O (60–90, 3 mL/min) to obtain **1** (28.8 mg) and **4** (4.2 mg). Fr.4.4 was separated by HPLC using MeOH/H_2_O (65–95, 3 mL/min) to obtain **2** (4.1 mg) and **3** (3.2 mg). Fr.4.5 was purified by HPLC (70–100% MeOH/H_2_O, 3 mL/min) to afford **5** (1.3 mg).

*Chrysogenones A* (**1**): White powder; [α]^20^_D_ + 206 (c 0.1, MeOH); UV (MeOH) λ_max_(log ε) 233 (2.01) nm, 282 (2.85) nm; ECD (MeOH) λ_max_ (Δε) 209 (+2.97) nm, 214 (−3.16) nm, 270 (+2.46) nm, 298 (−1.16) nm, 346 (+7.76) nm; ^1^H and ^13^C NMR data, see Table 1; HRESIMS *m/z* 551.2950 [M + Na]^+^ (calcd for C_31_H_44_O_7_Na^+^, 551.2985).

*Chrysogenones B* (**2**): White powder; [α]^20^_D_ + 234 (c 0.1, MeOH); UV (MeOH) λ_max_(log ε) 233 (2.07) nm, 281 (2.90) nm; ECD (MeOH) λ_max_ (Δε) 209 (+3.42) nm, 214 (−3.65) nm, 268 (+2.68) nm, 299 (−1.25) nm, 346 (+8.64) nm; ^1^H and ^13^C NMR data, see Table 1; HRESIMS *m/z* 565.3161 [M + Na]^+^ (calcd for C_32_H_46_O_7_Na^+^, 565.3141).

*Chrysogenones C* (**3**): White powder; [α]^20^_D_ + 281 (c 0.1, MeOH); UV (MeOH) λ_max_(log ε) 228 (2.17) nm, 282 (2.94) nm; ECD (MeOH) λ_max_ (Δε) 209 (+4.27) nm, 214 (−4.51) nm, 267 (+2.01) nm, 298 (−2.25) nm, 346 (+9.61) nm; ^1^H and ^13^C NMR data, see Table 1; HRESIMS *m/z* 465.3034 [M + Na]^+^ (calcd for C_28_H_42_O_4_Na^+^, 465.2981).

*Chrysogenones D* (**4**): White powder; [α]^20^_D_ + 286 (c 0.1, MeOH); UV (MeOH) λ_max_(log ε) 219 (2.24) nm, 232 (2.29) nm, 248 (2.17) nm, 339 (2.87) nm; ECD (MeOH) λ_max_ (Δε) 221 (+2.01) nm, 238 (−0.78) nm, 250 (+1.34) nm, 289 (−0.06) nm, 361 (+8.97) nm; ^1^H and ^13^C NMR data, see Table 2; HRESIMS *m/z* 425.3024 [M + H]^+^ (calcd for C_28_H_41_O_3_^+^, 425.3056).

*Chrysogenones E* (**5**): White powder; [α]^20^_D_ + 219 (c 0.1, MeOH); UV (MeOH) λ_max_(log ε) 221 (2.39) nm, 229 (2.40) nm, 248 (2.31) nm, 335 (2.91) nm; ECD (MeOH) λ_max_ (Δε) 221 (+2.56) nm, 238 (−0.03) nm, 250 (+0.81) nm, 304 (−0.44) nm, 361 (+8.57) nm; ^1^H and ^13^C NMR data, see Table 2; HRESIMS *m/z* 511.3004 [M + H]^+^ (calcd for C_31_H_43_O_6_^+^, 511.3060).

### 3.4. ECD Calculation

At room temperature, the experimental ECD spectra of compounds **1**–**5** in methanol at a concentration of 1.0 mg/mL were recorded by a Chirascan spectrometer. The calculated ECD spectra of compound **4** were calculated by the IEFPCM model in MeOH at the B3LYP/6-311G (d, p) level using time-dependent density functional theory (TD-DFT).

### 3.5. X-Ray Crystallographic Data

Experimental. Single colorless block-shaped crystals of **1** were used as supplied. A suitable crystal with dimensions 0.18 × 0.08 × 0.08 mm^3^ was selected. Single-crystal X-ray diffraction data were collected by a Bruker D8 Venture diffractometer with Cu Kα radiation (*λ* = 1.54178 Å). The crystal was kept at a steady T = 100(2) K during data collection. The structure was solved with the ShelXT structure program using Intrinsic Phasing and refined with ShelXL using full-matrix least-squares minimization on *F*^2^. The crystallographic data were deposited in the Cambridge Crystallographic Data Center (CCDC) with the deposition number 2537563.

Crystal data for **1**: C_31_H_44_O_7_, *M* = 528.66 g/mol, monoclinic, space group *P*2_1_ (no. 4), *a* = 11.891(2) Å, *b* = 39.192(8) Å, *c* = 13.238(3) Å, *α* = 90°, *β* = 107.940(30)°, *γ* = 90°, *V* = 5870.00(2) Å3, *T* = 100(2) K, *Z* = 8, *μ*(Cu K*α*) = 0.674 mm^−1^, 61339 reflections measured, 23147 independent reflections (*R_int_* = 0.0657). The final *R_1_* value was 0.0451 (*I* > 2*σ*(*I*)). The final *wR*(*F*^2^) value was 0.1034 (*I* > 2*σ*(*I*)). The final *R_1_* value was 0.0557 (all data). The final *wR*(*F*^2^) value was 0.1096 (all data). The goodness of fit on *F*^2^ was 1.041. Flack parameter = −0.01(7).

### 3.6. Cell Culture and Anti-Renal Fibrosis Activity Assays In Vitro

Normal rat kidney fibroblasts (NRK-49F) were maintained in DMEM supplemented with 10% FBS and 1% penicillin/streptomycin. For the screening of chrysogenones A–E (**1**–**5**) possessing anti-renal fibrotic activity, cells were seeded into 12-well plates. Following overnight incubation, the cells were serum-starved for 6 h and subsequently treated with 10 ng mL^−1^ recombinant TGF-β1 (PeproTech) for 48 h, either in the presence or absence of a candidate compound at the specified concentration.

### 3.7. Ability of Small-Molecule Compounds to Inhibit Proliferation of Myofibroblasts

The anti-proliferative effects of chrysogenones A–E (**1**–**5**) were evaluated on both TGF-β1-stimulated and unstimulated NRK-49F fibroblasts using a Cell Counting Kit-8 (CCK-8; Abbkine, Wuhan, China). Cells were seeded in 96-well plates at a density of 5·× 10^3^ cells per well and allowed to adhere overnight. For the TGF-β1 stimulated group, adherent cells were subjected to serum starvation for 6 h, followed by a 24 h exposure to 10 ng/mL recombinant human TGF-β1 in combination with specified concentrations of each compound. For the unstimulated group, cells were incubated for 24 h with specified concentrations of chrysogenones A–E alone. Subsequently, the CCK-8 reagent was then added, followed by a 1–2 h incubation at 37 °C in the dark. Absorbance was measured at 450 nm using a microplate reader (BioTek, Agilent, Winooski, VT, USA). Background absorbance from wells containing medium alone was subtracted as blank controls.

### 3.8. qPCR Analysis

Total RNA was isolated from NRK-49F cells with AG RNAex Pro reagent (Accurate Biology, Wuhan, China) following the manufacturer’s instructions. cDNA was synthesized from 1 μg total RNA using HiScript III RT SuperMix (Vazyme, Nanjing, China). Target gene expression was quantified by SYBR Green-based RT-qPCR, with data normalized to the reference gene HPRT1 and analyzed via the 2^−ΔΔCT^ method.

### 3.9. Western Blot Analysis

For immunoblotting, total protein lysates from NRK-49F cells were resolved by SDS-PAGE and electrotransferred onto PVDF membranes. Membranes were blocked in 5% skimmed milk prior to incubation with primary antibodies (4 °C, overnight) and corresponding secondary antibodies (room temperature, 1 h). Chemiluminescent detection was performed with ECL, and images were acquired on a Tanon 5200CE system. Densitometric analysis was conducted in ImageJ (Version 1.53a), with values normalized to the β-tubulin loading control. Primary antibodies included anti-Collagen I (Abcam, Cambridge, UK), anti-α-SMA (Cell Signaling Technology, Danvers, MA, USA), and anti-β-tubulin (Cell Signaling Technology, Danvers, MA, USA).

### 3.10. Network Pharmacology

We employed a bioinformatic workflow to identify potential targets of Compound 2 in renal fibrosis. Protein targets of compound **2** were predicted using SwissTargetPrediction, and known disease-associated targets were sourced from GeneCards. The intersecting target set was identified with the Venny 2.1 website. These overlapping targets were then submitted to the STRING database to construct a protein–protein interaction (PPI) network. The resulting network was imported into Cytoscape software (v3.9.1) for visualization and further analysis. Core targets were defined via the cytoHubba plugin based on the top 3 rankings in maximal node connect degree (Degree).

### 3.11. Molecular Docking

Molecular docking studies were performed to investigate the binding mode between compound **2** and the three putative target proteins encoded by AKT1, HSP90AA1, and MDM2 using Autodock Vina (v1.2.x). The three-dimensional (3D) coordinates of the AKT1 protein (PDB ID: 6HHG), HSP90AA1 protein (PDB ID: 3BM9), and MDM2 protein (PDB ID: 4OBA) were downloaded from the Protein Data Bank [http://www.rcsb.org (accessed on 27 January 2026)] and later prepared by removing water and adding hydrogen using PyMol 3.1.6.1 software. The 3D structure of compound **2** was obtained by ChemDraw Professional 17.0 and Chem3D 17.0 software. Docking input files were generated by the AutoDockTools 1.5.7 package. The search grid of the AKT1 protein was identified as center_x 13.6, center_y −11.8, center_z −15.8, with dimensions size_x 17.9, size_y = 17.9, size_z = 17.9; HSP90AA1 protein as center_x 0.5, center_y 30, center_z 20, with dimensions size_x 16.4, size_y 20.5, size_z 20.5; and MDM2 protein as center_x 26.8, center_y 17.5, center_z −3.1, with dimensions size_x 33.1, size_y 28.8, size_z 33.1. The best-scoring pose, as judged by the Vina docking score, was chosen and visually analyzed using PyMoL 3.1.6.1 software.

## 4. Conclusions

The chemical investigation of the deep-sea-derived *Penicillium* sp. MCCC 3A00121 led to the discovery of five previously undescribed metabolites, chrysogenones A–E (**1**–**5**), belonging to a rare class of oxidatively modified ergostane-type steroids. Among them, chrysogenones A–B, E (**1**–**2**, **5**) possessed an unusual malonyl moiety attached to the C-12 position of the ergostane core. Compounds **2**–**4** demonstrated varying degrees of inhibition of renal fibrosis, as indicated by the suppressed expression of the fibrotic biomarkers α-SMA and COL1A1. Of particular note, chrysogenone B (**2**) exhibited the most potent activity and effectively inhibited the proliferation of activated NRK-49F cells above 20 μM. Furthermore, integrative network pharmacology profiling coupled with molecular docking simulations suggested that the anti-fibrotic effects of compound **2** may involve modulation of key targets such as AKT1, HSP90AA1, and MDM2. However, further enzymatic investigations are still needed to validate these putative interactions and the regulatory relationship between the aforementioned proteins and the TGF-β signaling pathway in the near future. Such results will substantially enhance the robustness of our conclusions.

## Figures and Tables

**Table 1:** The ^1^H and ^13^C NMR spectroscopic data for chrysogenones A–C (**1**–**3**) (*δ* in ppm, *J* in Hz within the parentheses).

	1 ^a^		2 ^a^		3 ^a^	
**Pos.**	*δ*_H_	*δ*_C_	*δ*_H_	*δ*_C_	*δ*_H_	*δ*_C_
1	2.11, m	32.1, CH_2_	2.11, m	32.1, CH_2_	2.10, m	32.2, CH_2_
	2.26, m		2.26, m		2.26, m	
2	2.26, m	34.3, CH_2_	2.26, m	34.3, CH_2_	2.26, m	34.4, CH_2_
	2.50, m		2.50, m		2.51, m	
3		198.5, C		198.5, C		198.6, C
4	5.56, s	121.7, CH	5.56, s	121.7, CH	5.55, s	121.5, CH
5		165.5, C		165.4, C		166.0, C
6	6.43, d (9.9)	128.9, CH	6.44, d (9.9)	128.9, CH	6.40, d (9.8)	128.6, CH
7	6.33, d (9.9)	136.6, CH	6.34, d (9.9)	136.6, CH	6.30, d (9.8)	137.2, CH
8		72.3, C		72.2, C		72.6, C
9		76.4, C		76.4, C		76.3, C
10		41.0, C		41.0, C		41.0, C
11	1.87, m	36.6, CH_2_	1.87, m	36.6, CH_2_	1.76, m	41.0, CH_2_
	1.62, m		1.62, m		1.54, m	
12	5.04, dd (10.9, 4.4)	79.0, CH	5.06, dd (11.0, 4.4)	79.3, CH	3.64, m	74.0, CH
13		46.2, C		46.2, C		47.3, C
14	2.26, m	50.6, CH	2.20, m	50.5, CH	2.10, m	50.4, CH
15	1.56, m	19.1, CH_2_	1.57, m	19.1, CH_2_	1.48, m	19.2, CH_2_
	1.65, m		1.65, m		1.57, m	
16	1.69, m	23.7, CH2	1.68, m	23.5, CH_2_	1.60, m	24.8, CH_2_
	1.51, m		1.51, m		1.41, m	
17	1.48, m	56.6, CH	1.48, m	56.6, CH	1.37, m	57.4, CH
18	0.79 ^b^	9.7, CH_3_	0.78 ^b^	9.6, CH_3_	0.65, s	8.8, CH_3_
19	1.12, s	24.2, CH_3_	1.13, s	24.2, CH_3_	1.16, s	24.3, CH_3_
20	2.26, m	36.9, CH	2.21, m	36.8, CH	2.38, m	37.0, CH
21	0.86 ^c^	23.0, CH_3_	0.86 ^c^	23.0, CH_3_	1.05, d (7.3)	23.1, CH_3_
22	5.29, dd (15.3, 8.4)	133.9, CH	5.30, dd (15.3, 8.3)	133.9, CH	5.27, dd (14.8, 8.0)	135.1, CH
23	5.24, dd (15.3, 7.4)	132.2, CH	5.20, dd (15.3, 7.5)	132.3, CH	5.15, dd (14.8, 7.4)	131.4, CH
24	1.86, m	42.4, CH	1.85, m	42.4, CH	1.86, m	42.4, CH
25	1.46, m	32.6, CH	1.46, m	32.6, CH	1.45, m	32.6, CH
26	0.80, m	19.9, CH_3_	0.83 ^c^	19.9, CH_3_	0.81 ^b^	19.9, CH_3_
27	0.79 ^b^	19.6, CH_3_	0.78 ^b^	19.5, CH_3_	0.81 ^b^	19.6, CH_3_
28	0.89 ^c^	17.3, CH_3_	0.89 ^c^	17.4, CH_3_	0.89, d (7.2)	17.5, CH_3_
29		166.3, C		165.8, C		
30	3.32, m	42.1, CH_2_	3.47, m	41.6, CH_2_		
31		167.9, C		166.9, C		
32-OMe			3.62, s	52.1, CH_3_		
8-OH	4.60, s		4.62, s		4.38, s	
9-OH	4.35, s		4.37, s		4.09, s	
12-OH					4.21, d (6.0)	

^a^ Acquired in DMSO-*d*_6_; ^b,c^ Resonances with the same superscript within a column are overlapping.

**Table 2:** The ^1^H and ^13^C NMR spectroscopic data for chrysogenones D–E (**4**–**5**) (*δ* in ppm, *J* in Hz within the parentheses).

	4 ^a^		5 ^a^	
**Pos.**	*δ*_H_	*δ*_C_	*δ*_H_	*δ*_C_
1	2.33, m	27.0, CH_2_	2.36, m	27.0, CH_2_
	1.58, m		1.59, m	
2	2.42, m	33.7, CH_2_	2.44, m	33.6, CH_2_
	2.23, m		2.28, m	
3		198.2, C		198.2, C
4	5.65, s	124.4, CH	5.72, d (4.0)	124.7, CH
5		162.8, C		162.2, C
6	6.04, d (9.6)	124.5, CH	6.12, dd (9.7, 4.0)	125.1, CH
7	6.41, d (9.6)	130.8, CH	6.46, dd (9.7, 4.0)	130.3, CH
8		126.2, C		126.4, C
9		74.0, C		73.9, C
10		41.7, C		41.7, C
11	1.73, m	35.9, CH_2_	1.88, m	32.1, CH_2_
	1.50, m		1.72, m	
12	3.76, m	70.7, CH	5.19, m	76.4, CH
13		49.0, C		47.6, C
14		154.0, C		152.3, C
15	1.65, m	22.8, CH_2_	1.75, m	21.9, CH_2_
	1.61, m		1.62, m	
16	2.39, m	25.1, CH_2_	2.49, m	24.8, CH_2_
	2.22, m		2.39, m	
17	1.46, m	55.8, CH	1.62, m	54.9, CH
18	0.80 ^b^	13.2, CH_3_	0.95 ^b^	14.1, CH_3_
19	0.94, s	20.4, CH_3_	0.91 ^b^	23.4, CH_3_
20	2.80, m	35.8, CH	2.52, m	35.9, CH
21	1.01, d (6.9)	23.2, CH_3_	0.99, m	20.3, CH_3_
22	5.33, dd (15.3, 8.4)	134.0, CH	5.32, m	133.0, CH
23	5.18, dd (15.3, 8.4)	132.3, CH	5.32, m	132.9, CH
24	1.84, q (6.8)	42.5, CH	1.87, m	42.4, CH
25	1.42, m	32.6, CH	1.47, m	32.6, CH
26	0.77 ^b^	20.0, CH_3_	0.82 ^c^	20.0, CH_3_
27	0.77 ^b^	19.6, CH_3_	0.82 ^c^	19.6, CH_3_
28	0.87, d (6.8)	17.5, CH_3_	0.89, d (6.9)	17.2, CH_3_
29				166.2, C
30			3.39, m	42.1, CH_2_
31				167.9, C
9-OH	4.41, s		4.78, s	
12-OH	4.52, d (5.1)			

^a^ Acquired in DMSO-*d*_6_; ^b,c^ Resonances with the same superscript within a column are overlapping.

## Data Availability

The data presented in this study are available in Supplementary Materials.

## Supplementary Materials

The following supporting information can be downloaded at https://www.mdpi.com/article/10.3390/md24030121/s1. Figures S1–S35. NMR, HRMS, HSQC, COSY, HMBC and NOESY spectrums of compounds **1**−**5**; Figure S36. cytotoxicity data of **1**−**5**.
