# Supplementary material for: Chrysogenones A–E: Malonyl-Modified Ergosterone Derivatives from Deep-Sea-Derived Penicillium sp. MCCC 3A00121 as Inhibitors of Renal Fibroblast Activation
Source: Mar Drugs. 2026 Mar 23;24(3):121. doi: 10.3390/md24030121 (PMC13027500; doi:10.3390/md24030121)

Supporting information

Chrysogenones A–E: Malonyl-Modified Ergosterone Derivatives from Deep-Sea-Derived *Penicillium* sp. MCCC 3A00121 as Inhibitors of Renal Fibroblast Activation

Content

Figure S1. HRESI mass spectrum of compound **1**;

Figure S2. ^{1}H NMR (400 MHz, DMSO-d_6) spectrum of compound **1**;

Figure S3. ^{13}C NMR (100 MHz, DMSO-d_6) spectrum of compound **1**;

Figure S4. HSQC spectrum of compound **1**;

Figure S5. COSY spectrum of compound **1**;

Figure S6. HMBC spectrum of compound **1**;

Figure S7. NOESY spectrum of compound **1**;

Figure S8. HRESI mass spectrum of compound **2**;

Figure S9. ^{1}H NMR (400 MHz, DMSO-d_6) spectrum of compound **2**;

Figure S10. ^{13}C NMR (100 MHz, DMSO-d_6) spectrum of compound **2**;

Figure S11. HSQC spectrum of compound **2**;

Figure S12. COSY spectrum of compound **2**;

Figure S13. HMBC spectrum of compound **2**;

Figure S14. NOESY spectrum of compound **2**;

Figure S15. HRESI mass spectrum of compound **3**;

Figure S16. ^{1}H NMR (400 MHz, DMSO-d_6) spectrum of compound **3**;

Figure S17. ^{13}C NMR (100 MHz, DMSO-d_6) spectrum of compound **3**;

Figure S18. HSQC spectrum of compound **3**;

Figure S19. COSY spectrum of compound **3**;

Figure S20. HMBC spectrum of compound **3**;

Figure S21. NOESY spectrum of compound **3**;

Figure S22. HRESI mass spectrum of compound **4**;

Figure S23. ^{1}H NMR (600 MHz, DMSO-d_6) spectrum of compound **4**;

Figure S24. ^{13}C NMR (150 MHz, DMSO-d_6) spectrum of compound **4**;

Figure S25. HSQC spectrum of compound **4**;

Figure S26. COSY spectrum of compound **4**;

Figure S27. HMBC spectrum of compound **4**;

Figure S28. NOESY spectrum of compound **4**;

Figure S29. HRESI mass spectrum of compound **5**;

Figure S30. ^{1}H NMR (400 MHz, DMSO-d_6) spectrum of compound **5**;

Figure S31. ^{13}C NMR (100 MHz, DMSO-d_6) spectrum of compound **5**;

Figure S32. HSQC spectrum of compound **5**;

Figure S33. COSY spectrum of compound **5**;

Figure S34. HMBC spectrum of compound **5**;

Figure S35. NOESY spectrum of compound **5**;

Figure S36. Cytotoxicity of chrysogenones A–E on unstimulated NRK-49F fibroblasts

Figure S1. HRESI mass spectrum of compound **1**.

Elemental Composition Report

Page 1

Single Mass Analysis

Tolerance = 50.0 mDa / DBE: min = -1.5, max = 50.0
Element prediction: Off
Number of isotope peaks used for i-FIT = 3

Monoisotopic Mass, Even Electron Ions
25 formula(e) evaluated with 6 results within limits (up to 50 closest results for each mass)
Elements Used:
C: 0-40 H: 0-50 O: 2-7 Na: 1-1
ZQB-28RE 1 (0.032)
1: TOF MS ES+

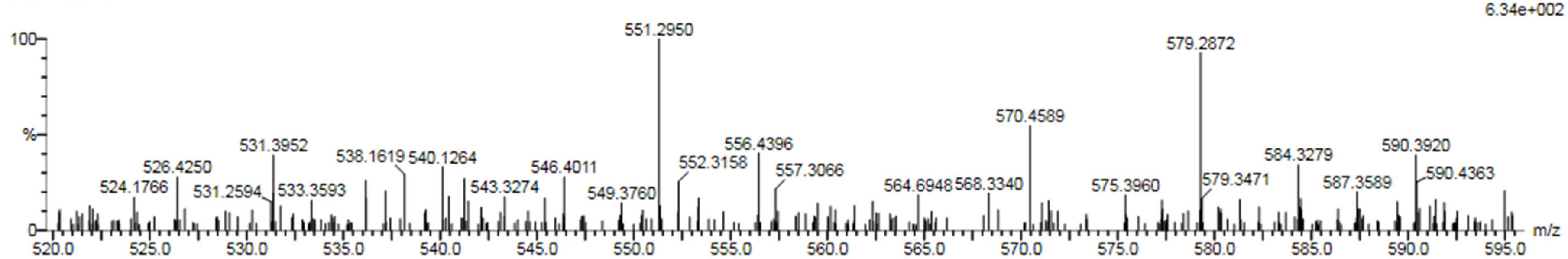

Minimum:				-1.5				
Maximum:		50.0	10.0	50.0				

Mass	Calc. Mass	mDa	PPM	DBE	i-FIT	Norm	Conf(%)	Formula
551.2950	551.2985	-3.5	-6.3	9.5	66.9	1.307	27.07	C31 H44 O7 Na
	551.3349	-39.9	-72.4	8.5	67.1	1.535	21.55	C32 H48 O6 Na
	551.2773	17.7	32.1	14.5	67.4	1.764	17.13	C34 H40 O5 Na
	551.3137	-18.7	-33.9	13.5	67.5	1.941	14.36	C35 H44 O4 Na
	551.2562	38.8	70.4	19.5	67.9	2.255	10.48	C37 H36 O3 Na
	551.2926	2.4	4.4	18.5	68.0	2.365	9.40	C38 H40 O2 Na

Figure S2. ^{1}H NMR (400 MHz, DMSO-d_6) spectrum of compound **1**.

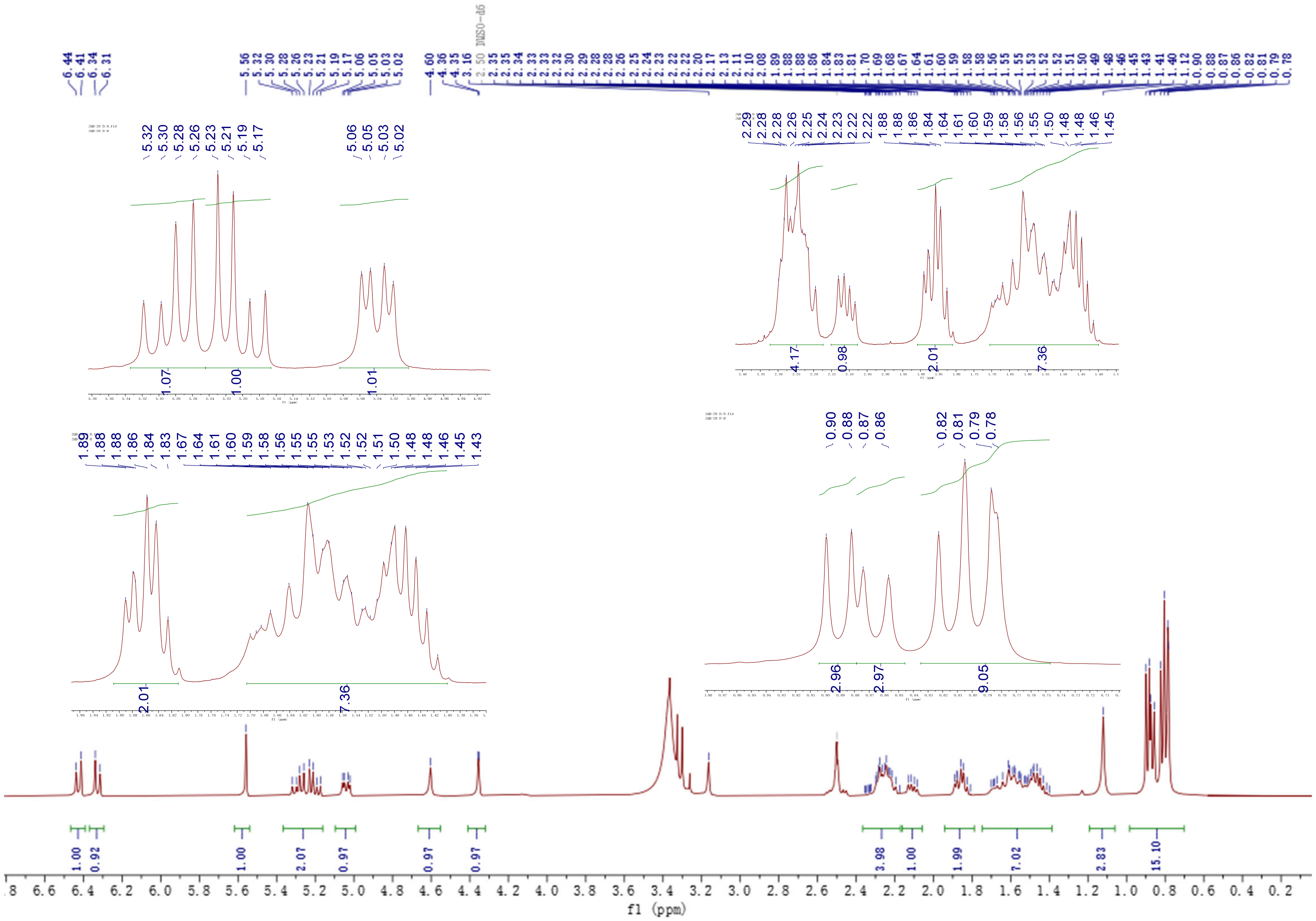

Figure S3. ^{13}C NMR (100 MHz, DMSO-d_6) spectrum of compound **1**.

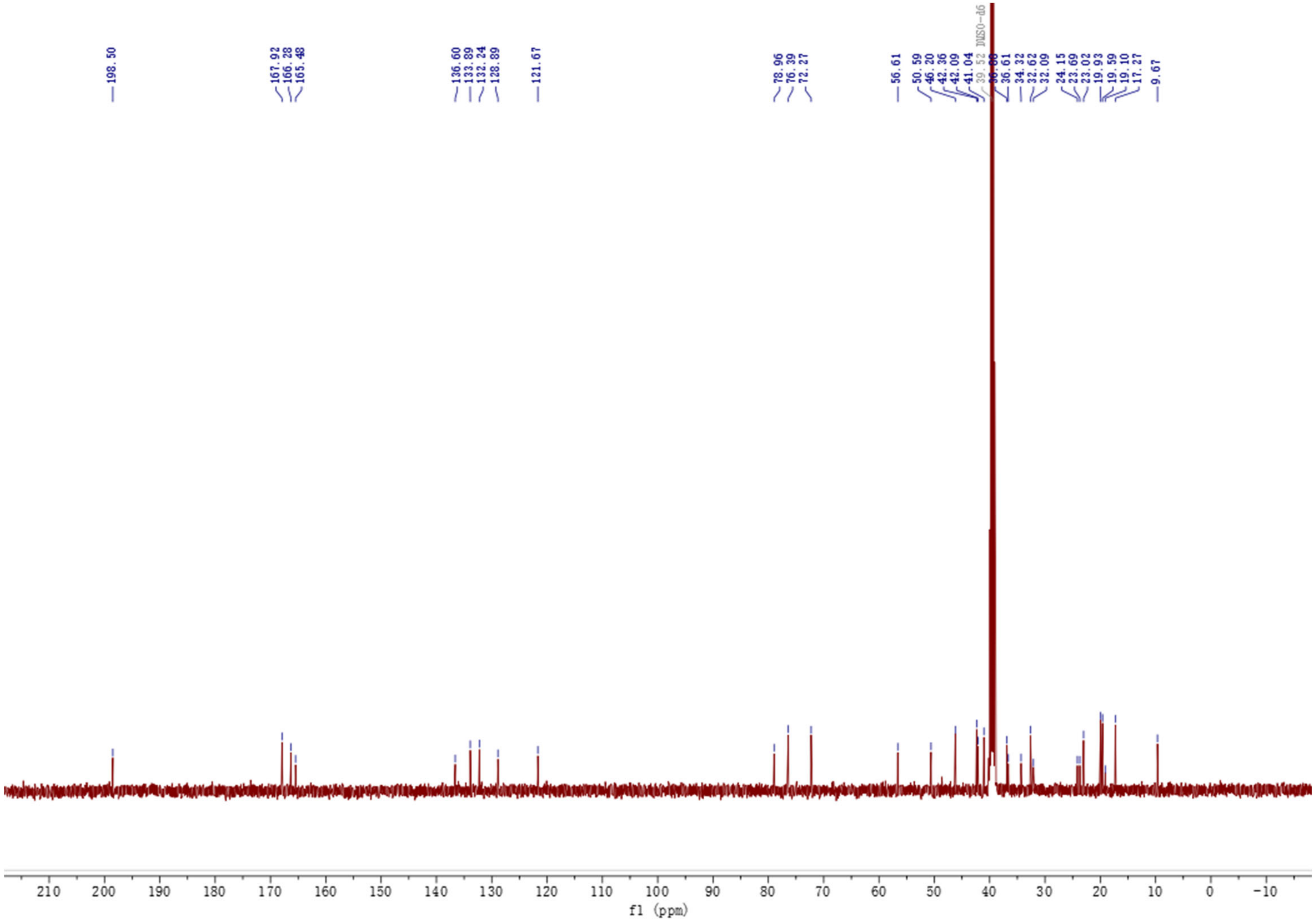

Figure S4. HSQC spectrum of compound **1**.

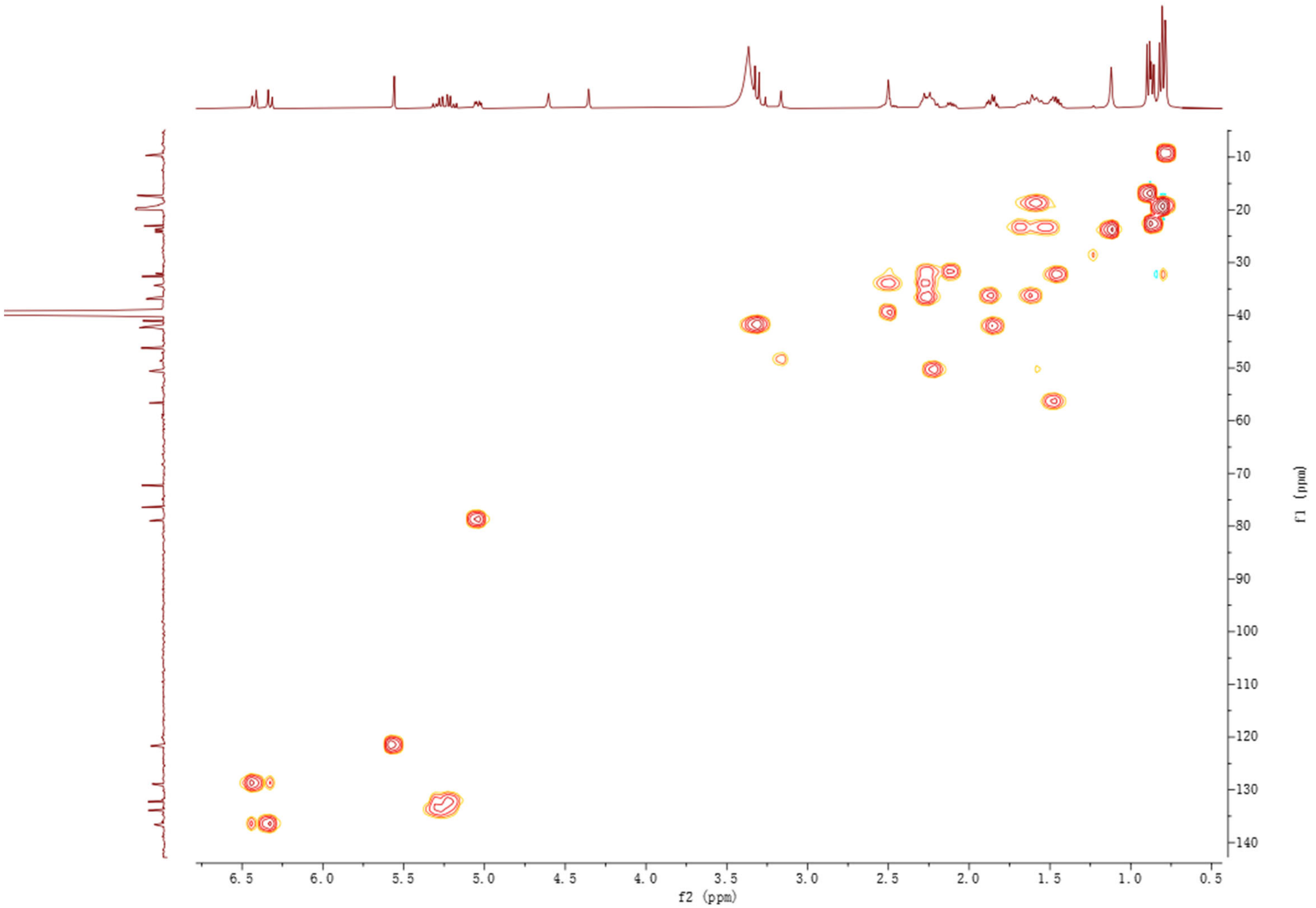

Figure S5. COSY spectrum of compound **1**.

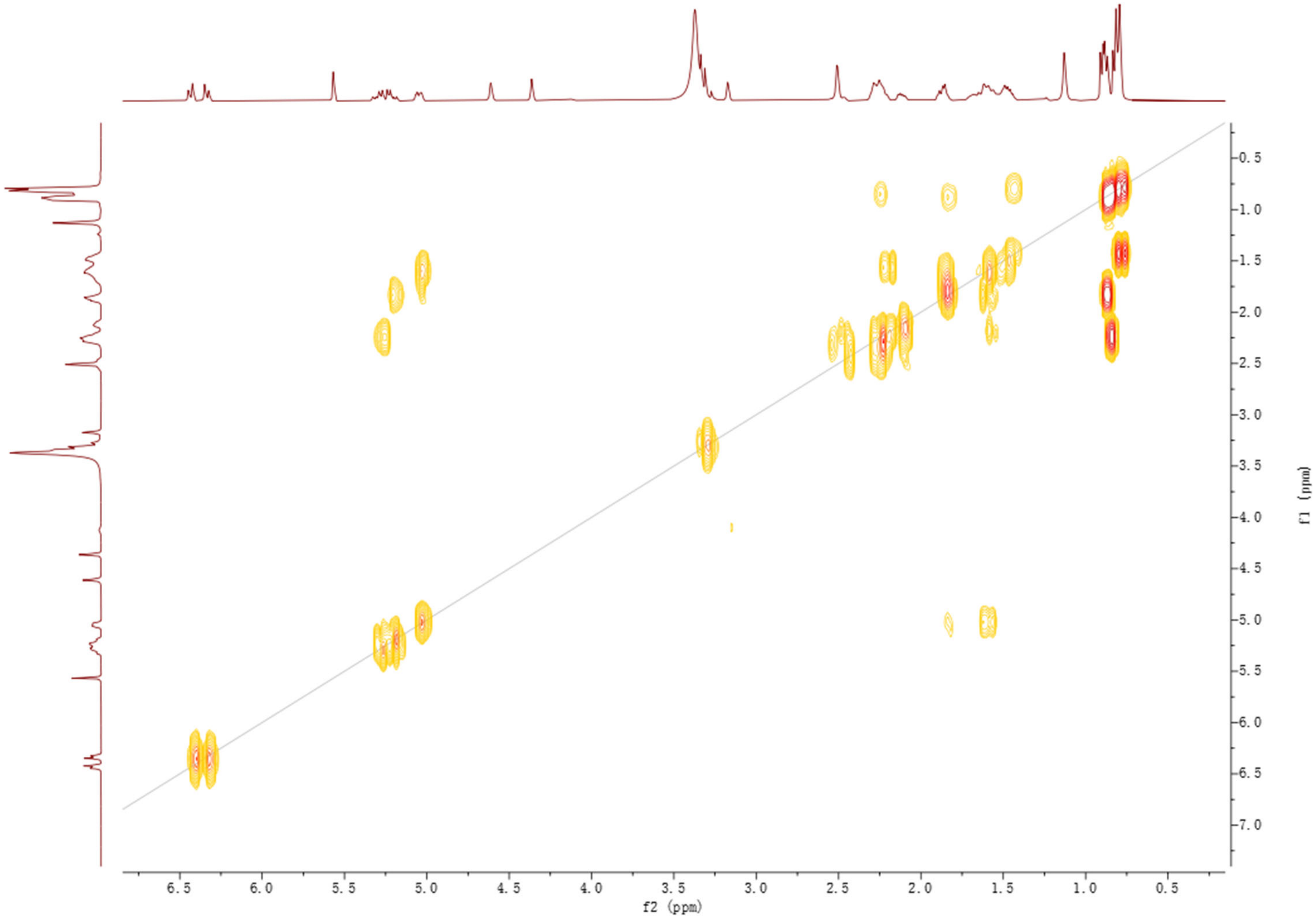

Figure S6. HMBC spectrum of compound **1**.

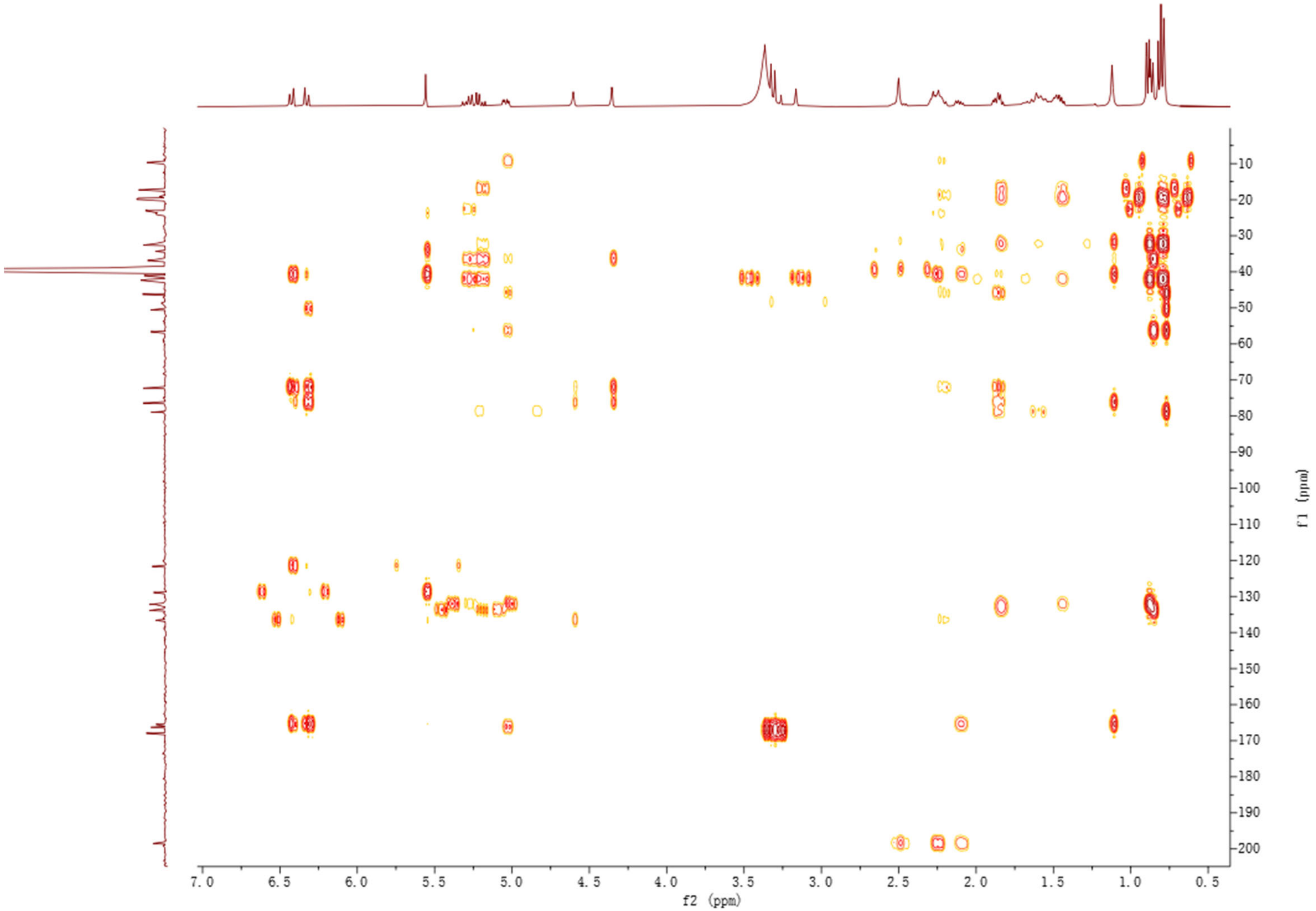

Figure S7. NOESY spectrum of compound **1**.

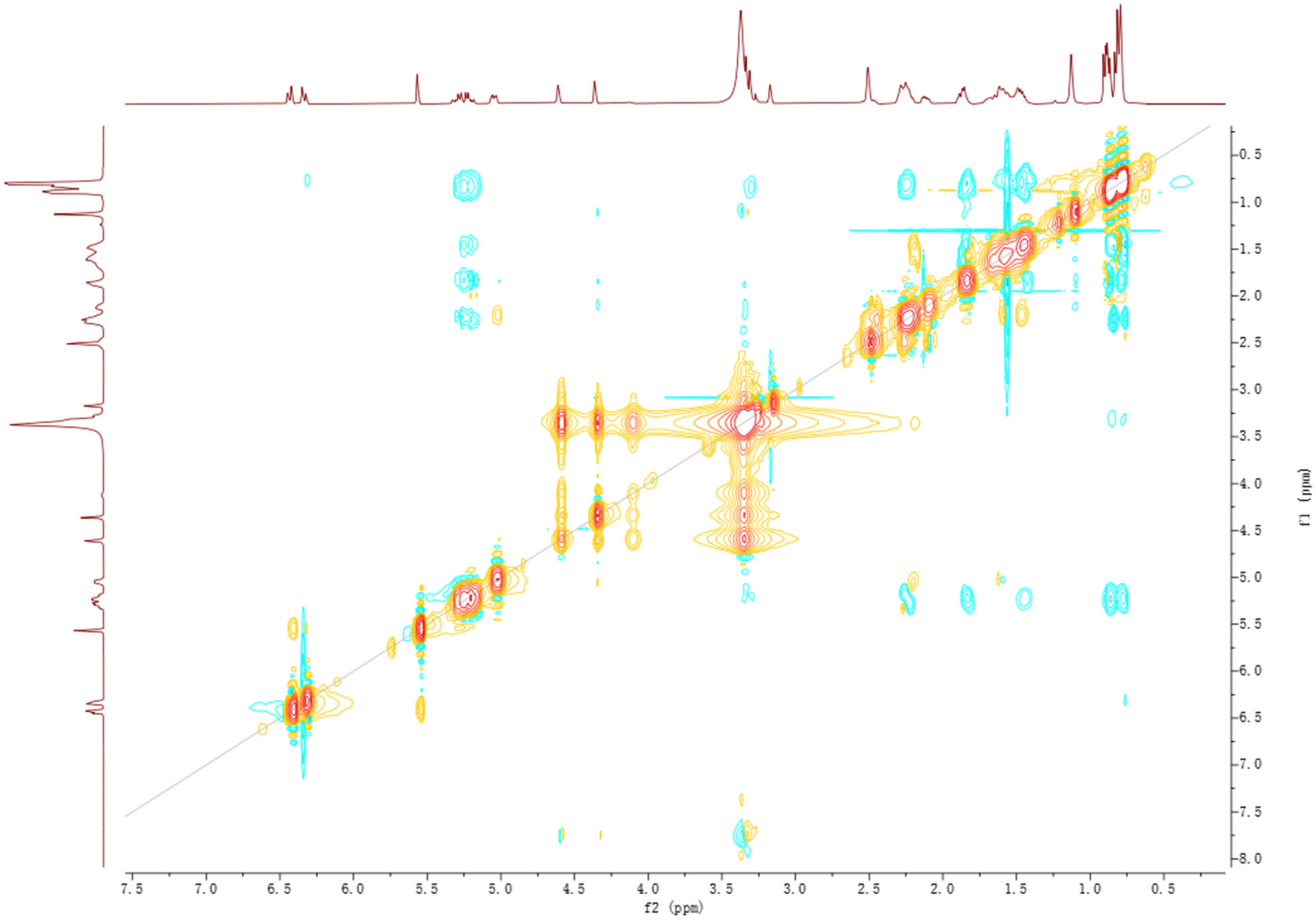

Figure S8. HRESI mass spectrum of compound **2**.

Elemental Composition Report

Page 1

Single Mass Analysis

Tolerance = 50.0 mDa / DBE: min = -1.5, max = 50.0
Element prediction: Off
Number of isotope peaks used for i-FIT = 3

Monoisotopic Mass, Even Electron Ions
24 formula(e) evaluated with 8 results within limits (up to 50 closest results for each mass)
Elements Used:
C: 0-40 H: 0-50 O: 0-7 Na: 1-1

ZQB-24 1 (0.032)
1: TOF MS ES+

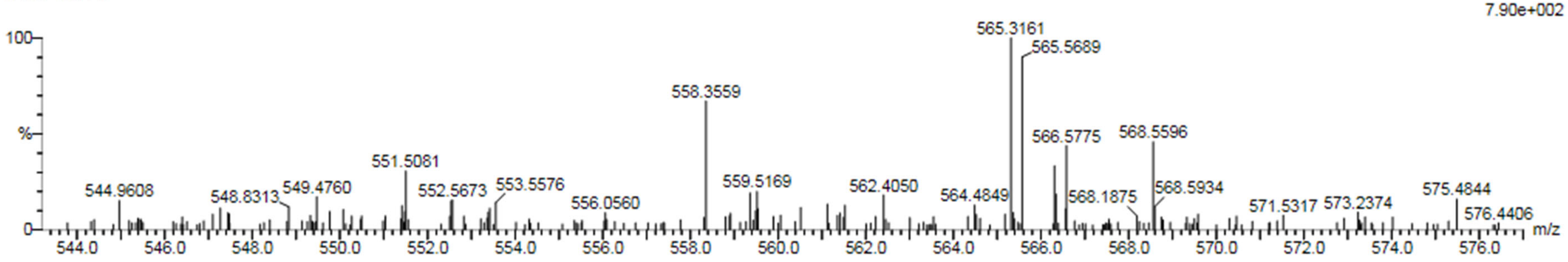

Minimum:				-1.5				
Maximum:		50.0	10.0	50.0				

Mass	Calc. Mass	mDa	PPM	DBE	i-FIT	Norm	Conf(%)	Formula
565.3161	565.3141	2.0	3.5	9.5	181.3	1.408	24.46	C32 H46 O7 Na
	565.3083	7.8	13.8	18.5	182.4	2.442	8.70	C39 H42 O2 Na
	565.3294	-13.3	-23.5	13.5	181.9	1.982	13.78	C36 H46 O4 Na
	565.2930	23.1	40.9	14.5	189.3	9.406	0.01	C35 H42 O5 Na
	565.3446	-28.5	-50.4	17.5	181.8	1.916	14.72	C40 H46 O Na
	565.3505	-34.4	-60.9	8.5	181.4	1.529	21.67	C33 H50 O6 Na
	565.2719	44.2	78.2	19.5	190.3	10.409	0.00	C38 H38 O3 Na
	565.3658	-49.7	-87.9	12.5	181.7	1.793	16.65	C37 H50 O3 Na

Figure S9. ^{1}H NMR (400 MHz, DMSO-d_6) spectrum of compound **2**.

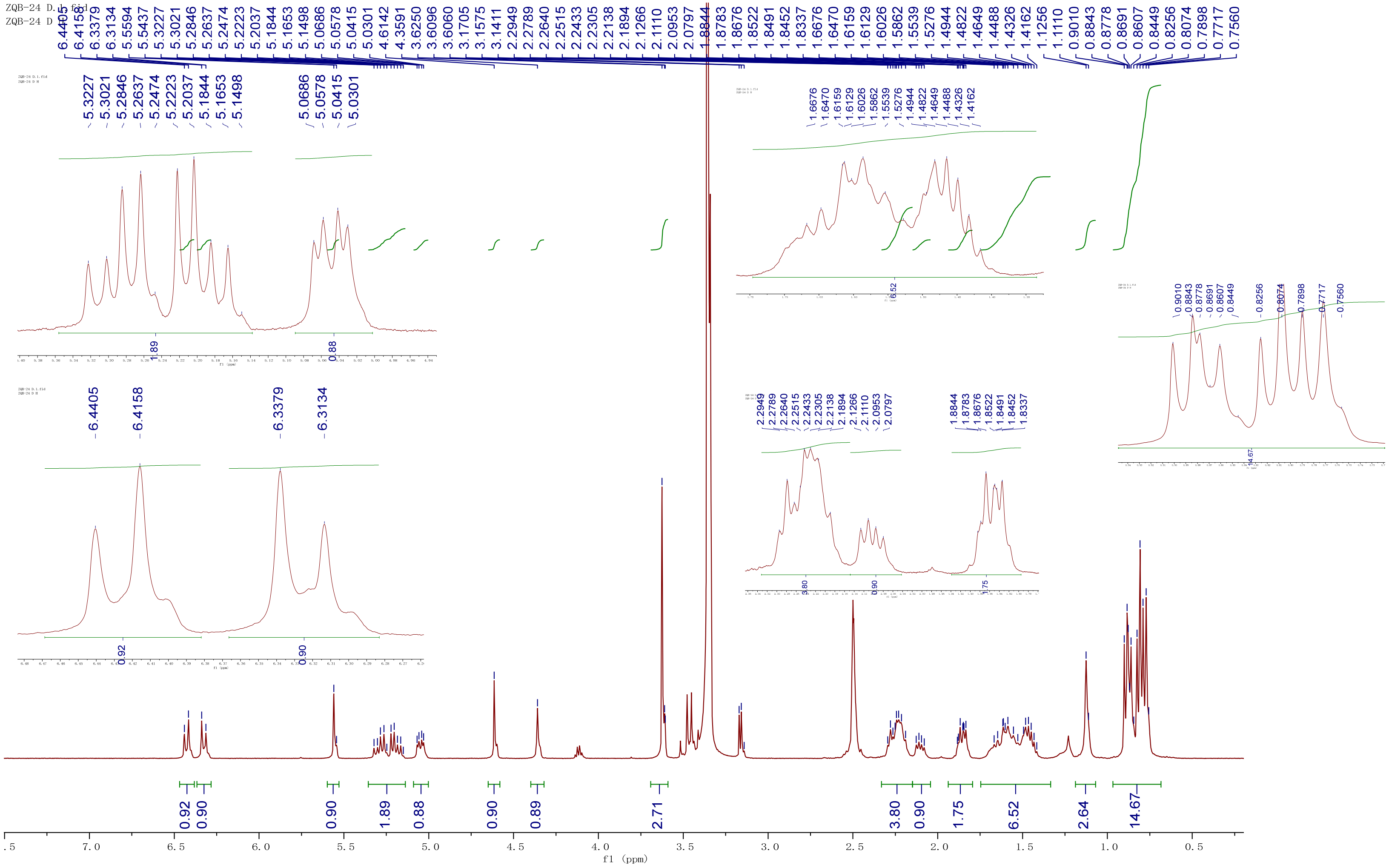

Figure S10. ^{13}C NMR (100 MHz, DMSO-d_6) spectrum of compound **2**.

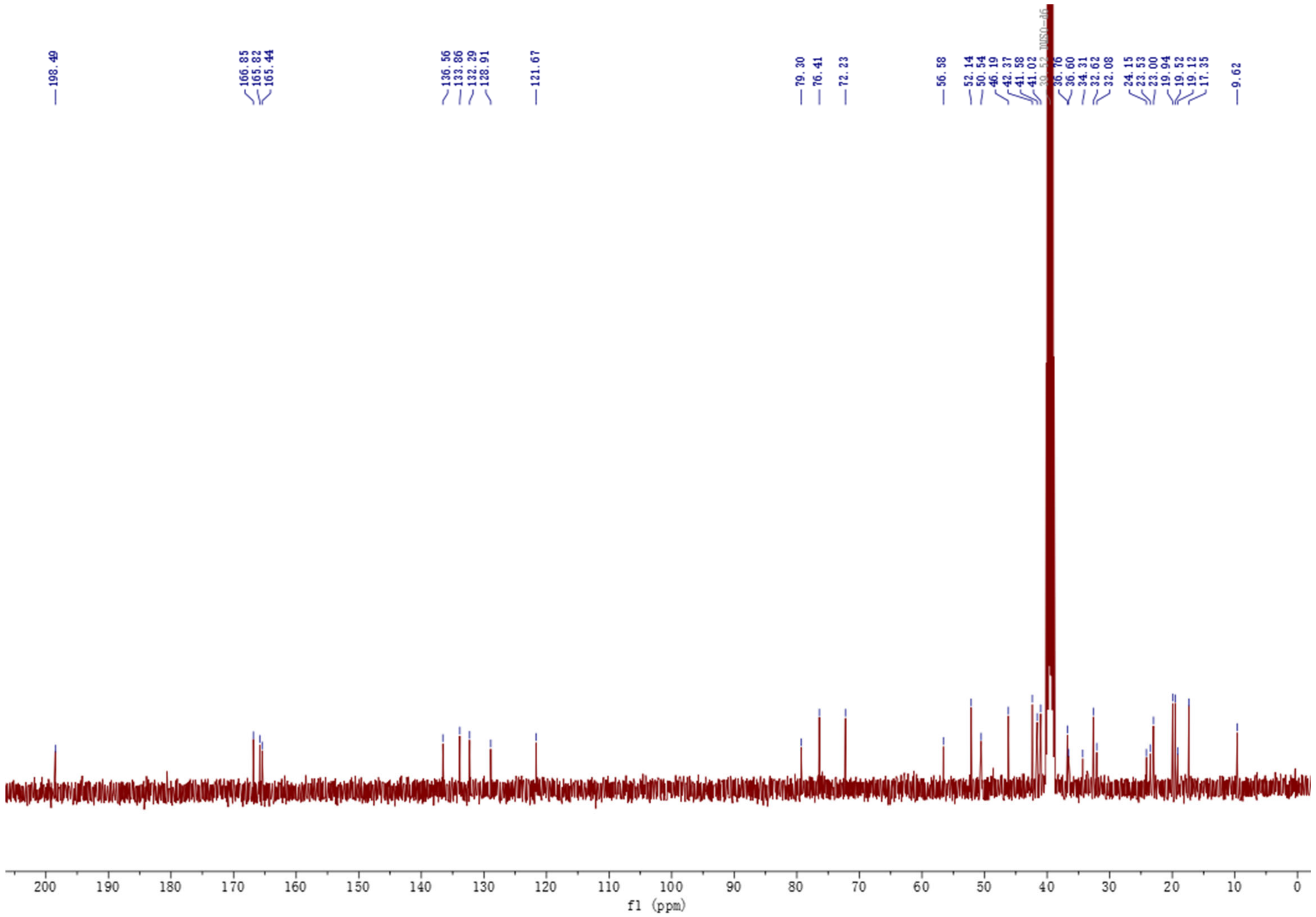

Figure S11. HSQC spectrum of compound **2**.

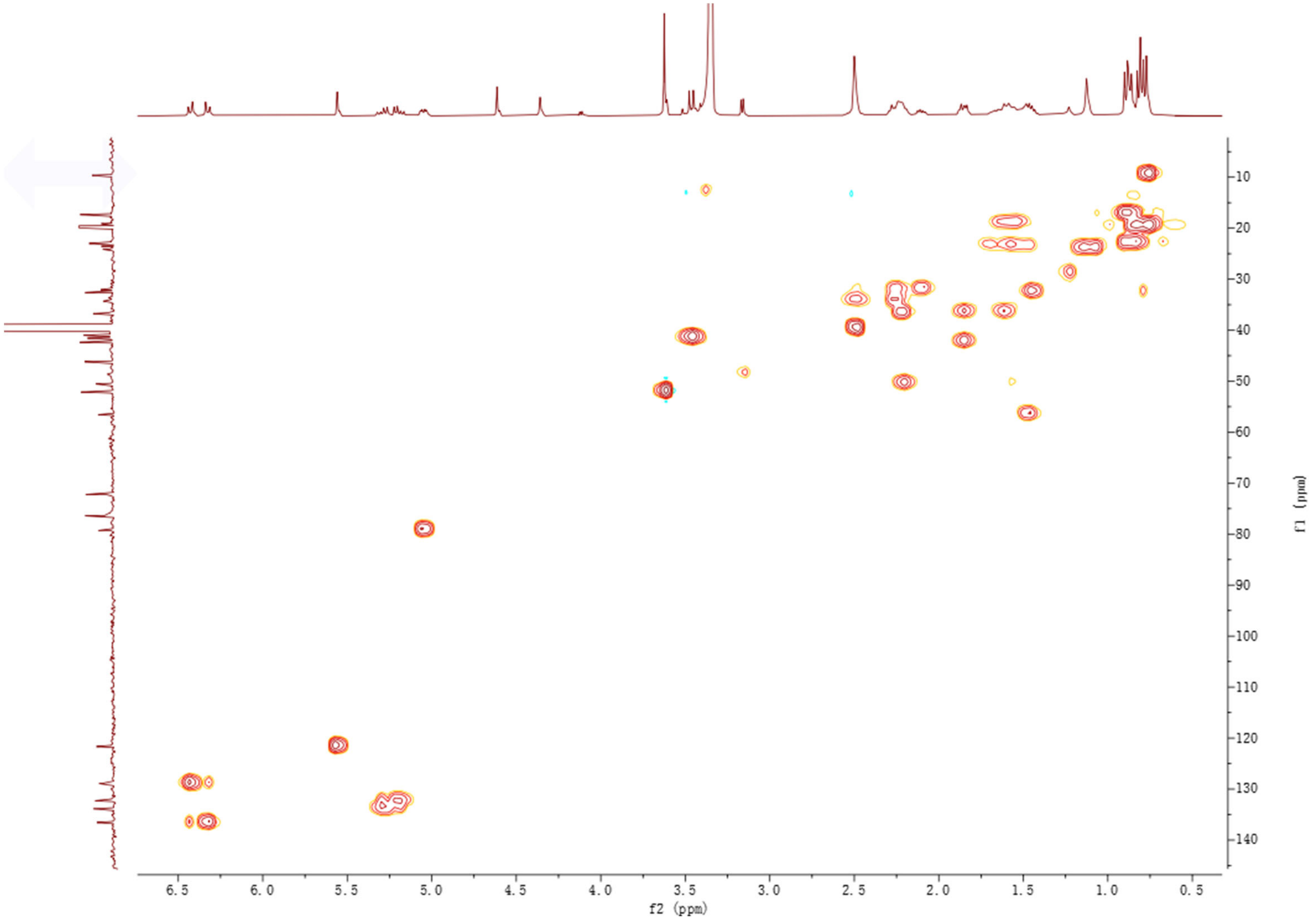

Figure S12. COSY spectrum of compound **2**.

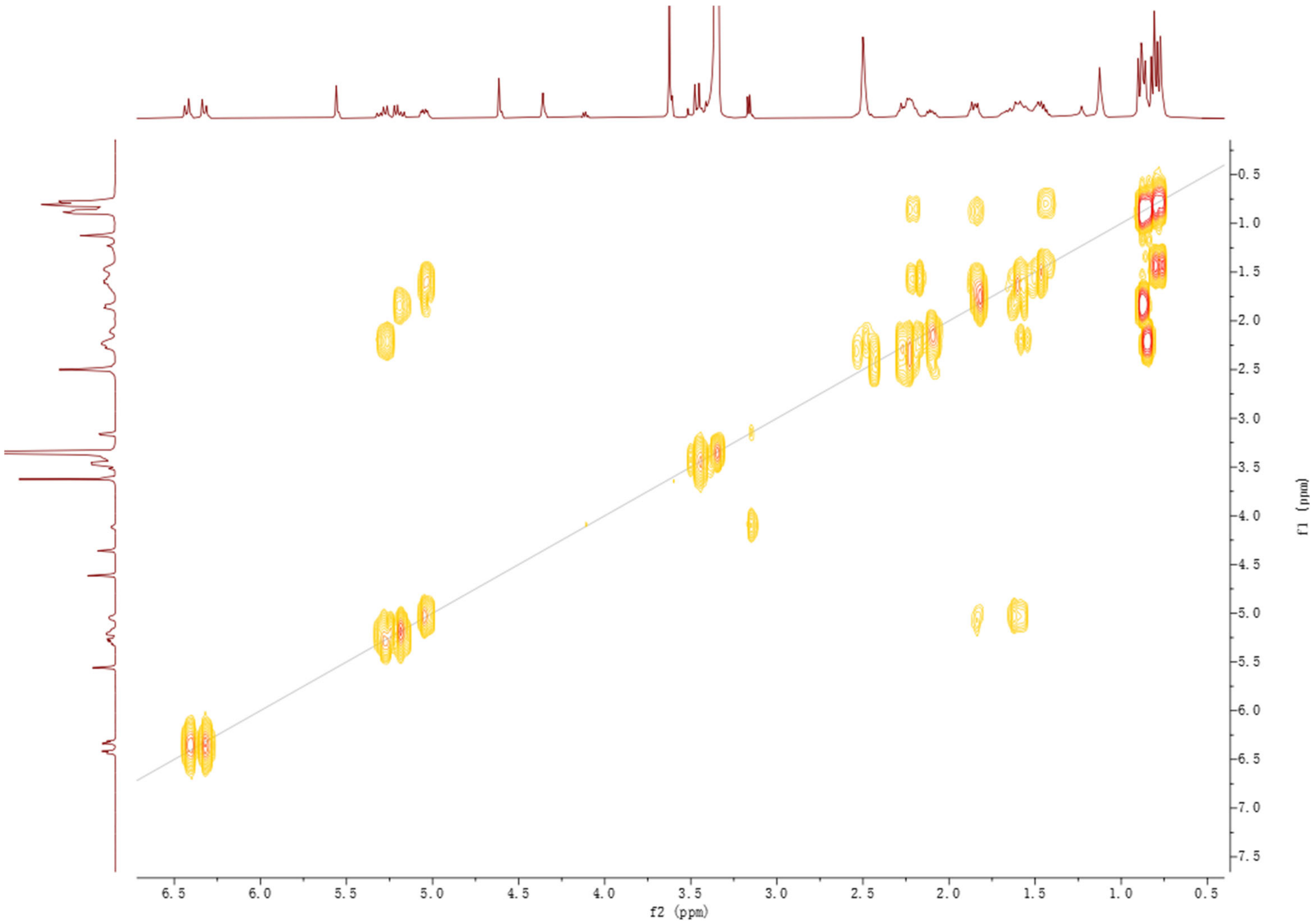

Figure S13. HMBC spectrum of compound **2**.

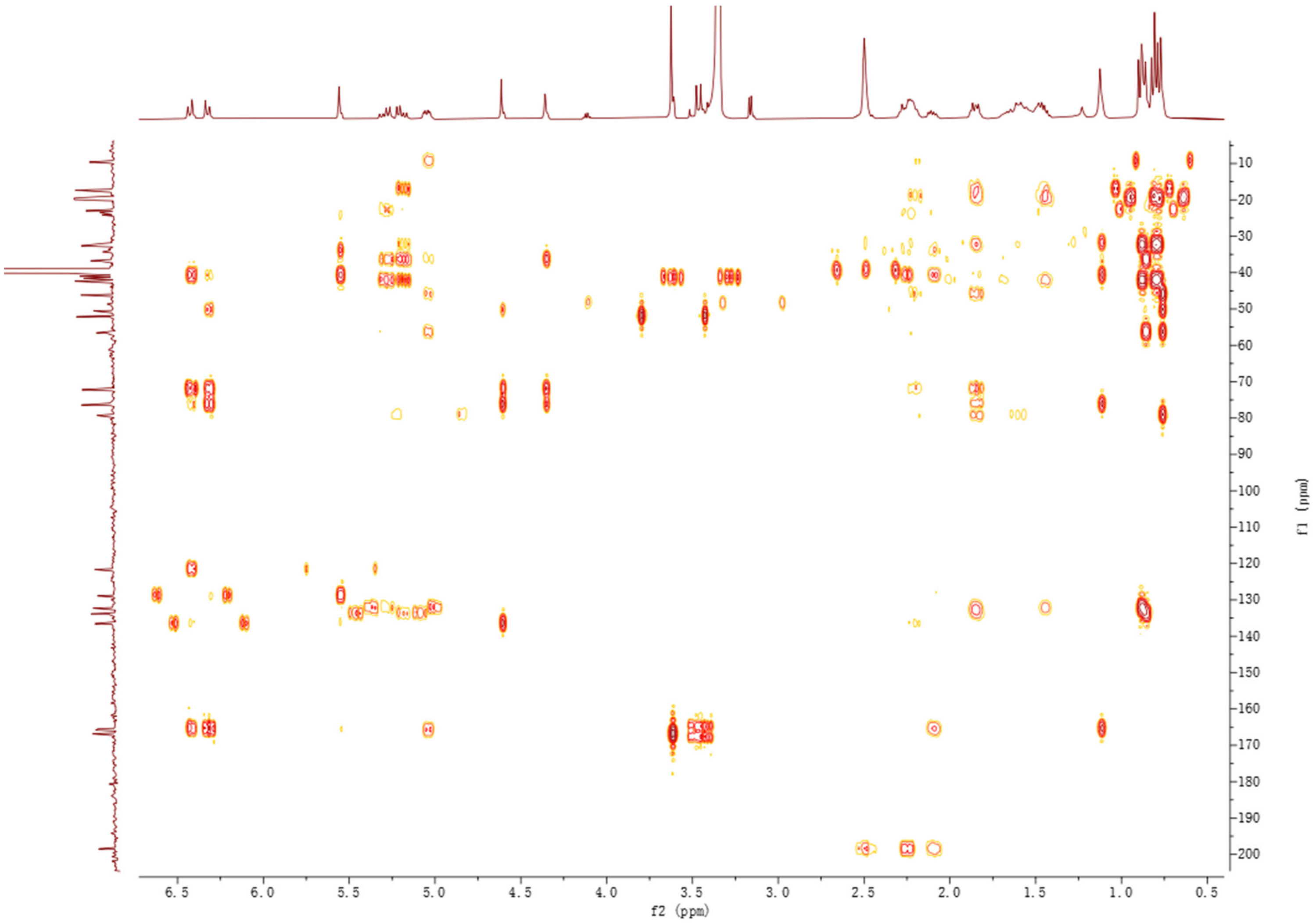

Figure S14. NOESY spectrum of compound **2**.

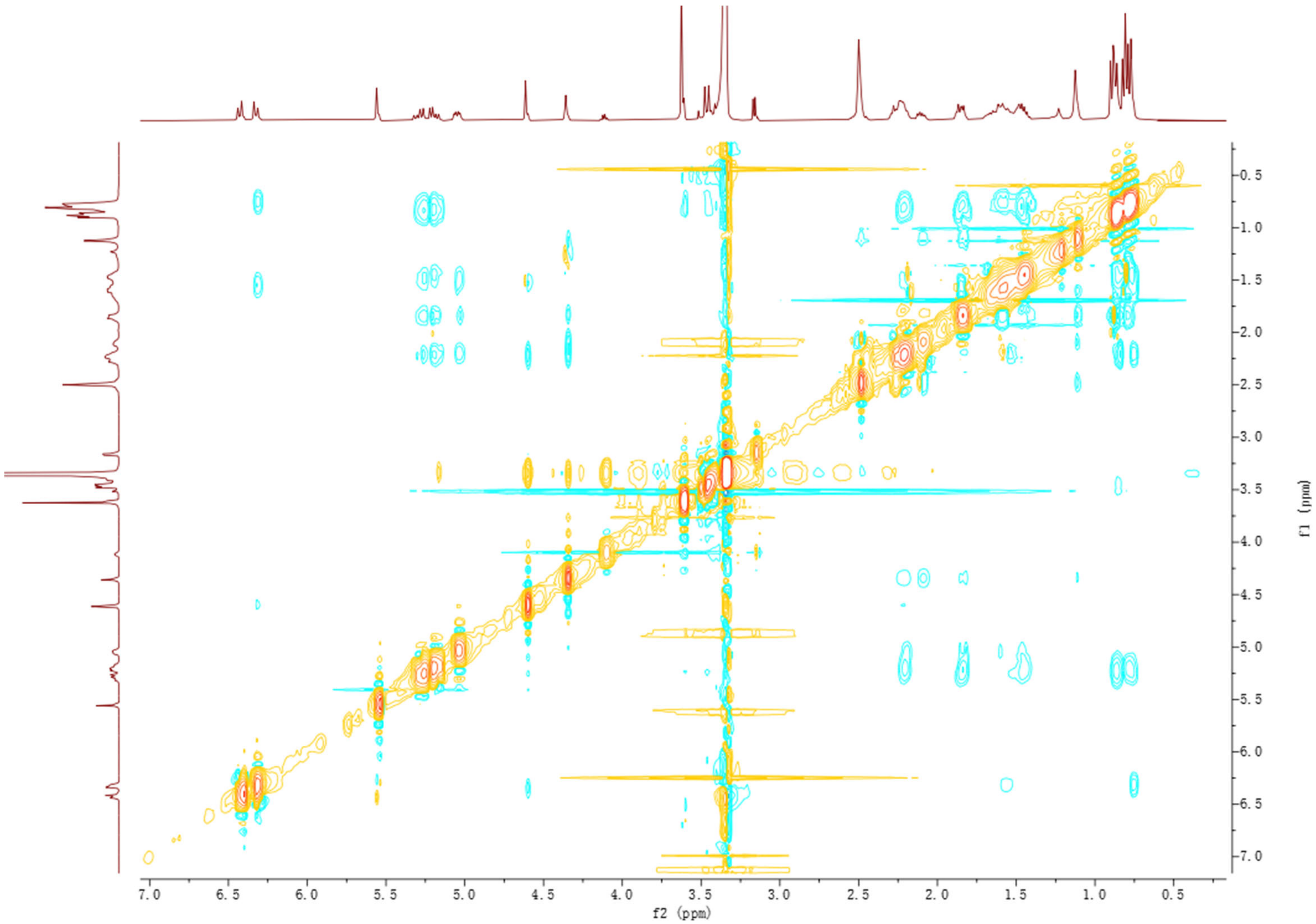

Figure S15. HRESI mass spectrum of compound **3**.

Elemental Composition Report

Single Mass Analysis

Tolerance = 50.0 mDa / DBE: min = -1.5, max = 50.0
Element prediction: Off
Number of isotope peaks used for i-FIT = 3

Monoisotopic Mass, Even Electron Ions
26 formula(e) evaluated with 6 results within limits (up to 50 closest results for each mass)
Elements Used:
C: 0-40 H: 0-50 O: 2-7 Na: 1-1
ZQB-27RE 1 (0.032)
1: TOF MS ES+

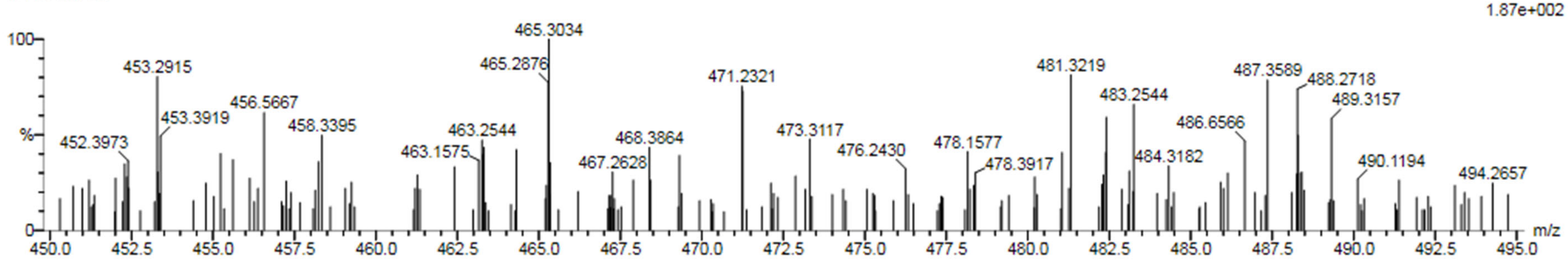

Minimum:				-1.5				
Maximum:		50.0	10.0	50.0				

Mass	Calc. Mass	mDa	PPM	DBE	i-FIT	Norm	Conf(%)	Formula
465.3034	465.2981	5.3	11.4	7.5	101.7	5.472	0.42	C28 H42 O4 Na
	465.3192	-15.8	-34.0	2.5	101.9	5.651	0.35	C25 H46 O6 Na
	465.2828	20.6	44.3	3.5	96.9	0.709	49.21	C24 H42 O7 Na
	465.2770	26.4	56.7	12.5	97.9	1.742	17.52	C31 H38 O2 Na
	465.3345	-31.1	-66.8	6.5	102.3	6.108	0.22	C29 H46 O3 Na
	465.2617	41.7	89.6	8.5	97.3	1.131	32.27	C27 H38 O5 Na

Figure S16. ^{1}H NMR (400 MHz, DMSO-d_6) spectrum of compound **3**.

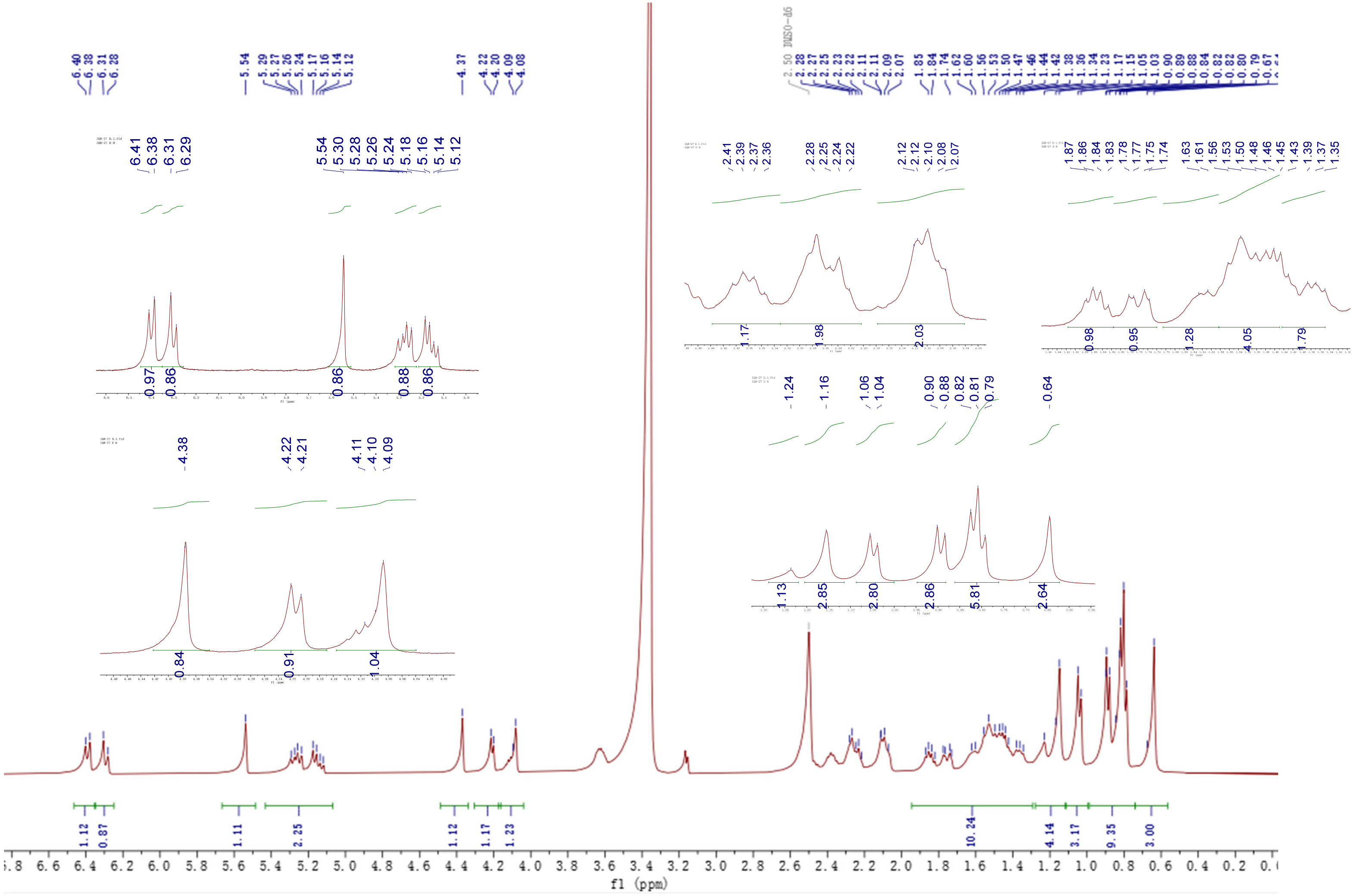

Figure S17. ^{13}C NMR (100 MHz, DMSO-d_6) spectrum of compound **3**.

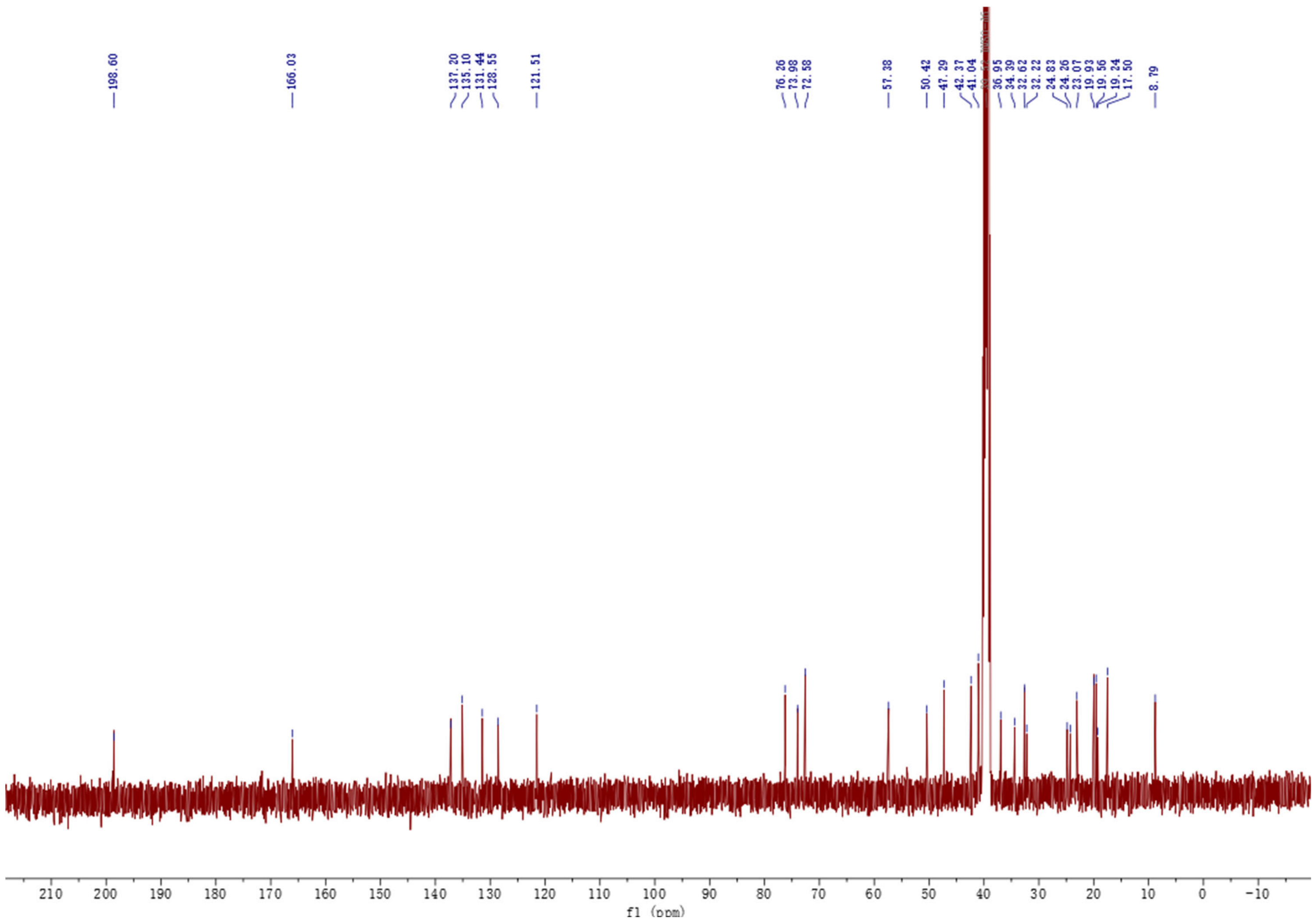

Figure S18. HSQC spectrum of compound **3**

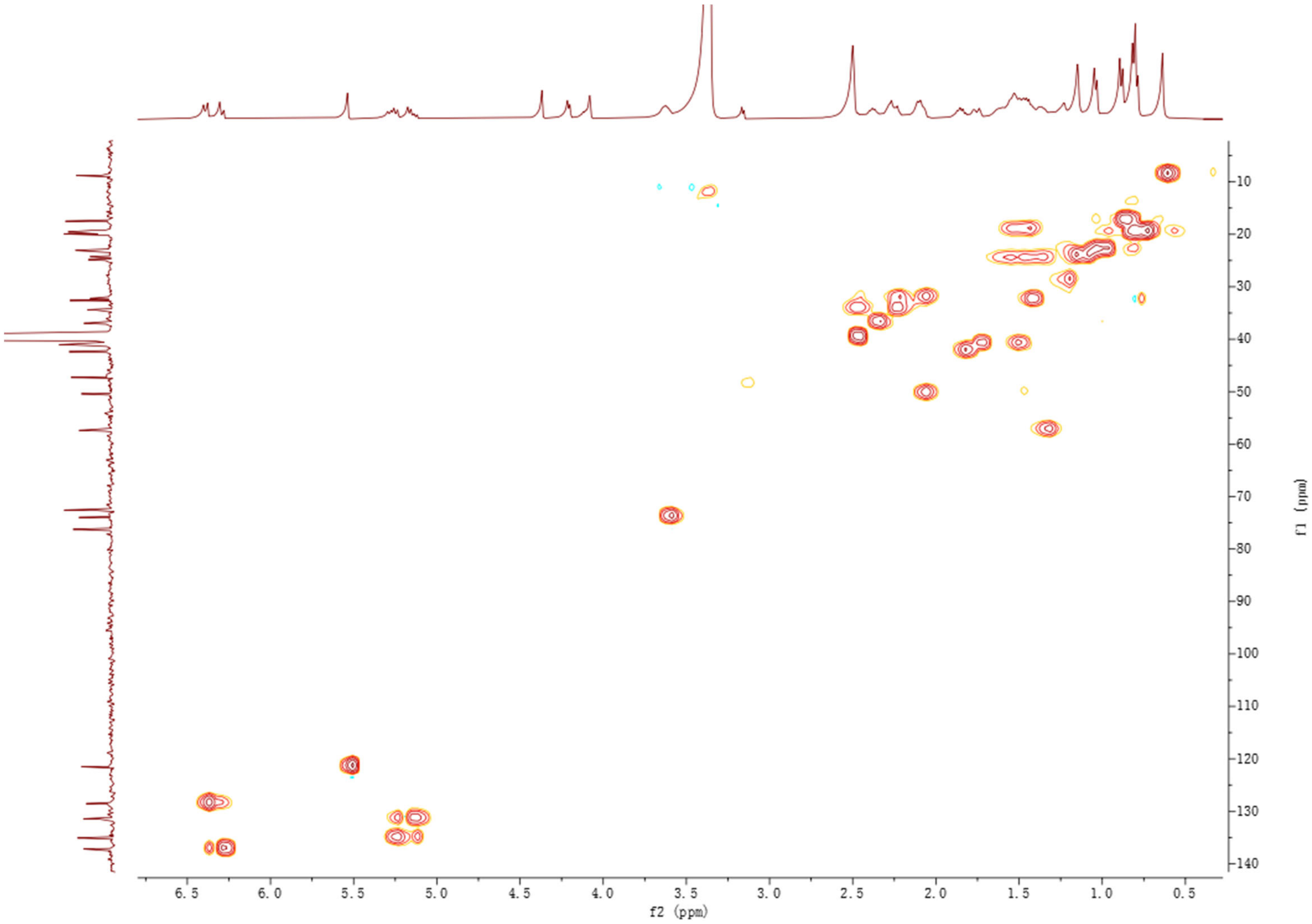

Figure S19. COSY spectrum of compound **3**.

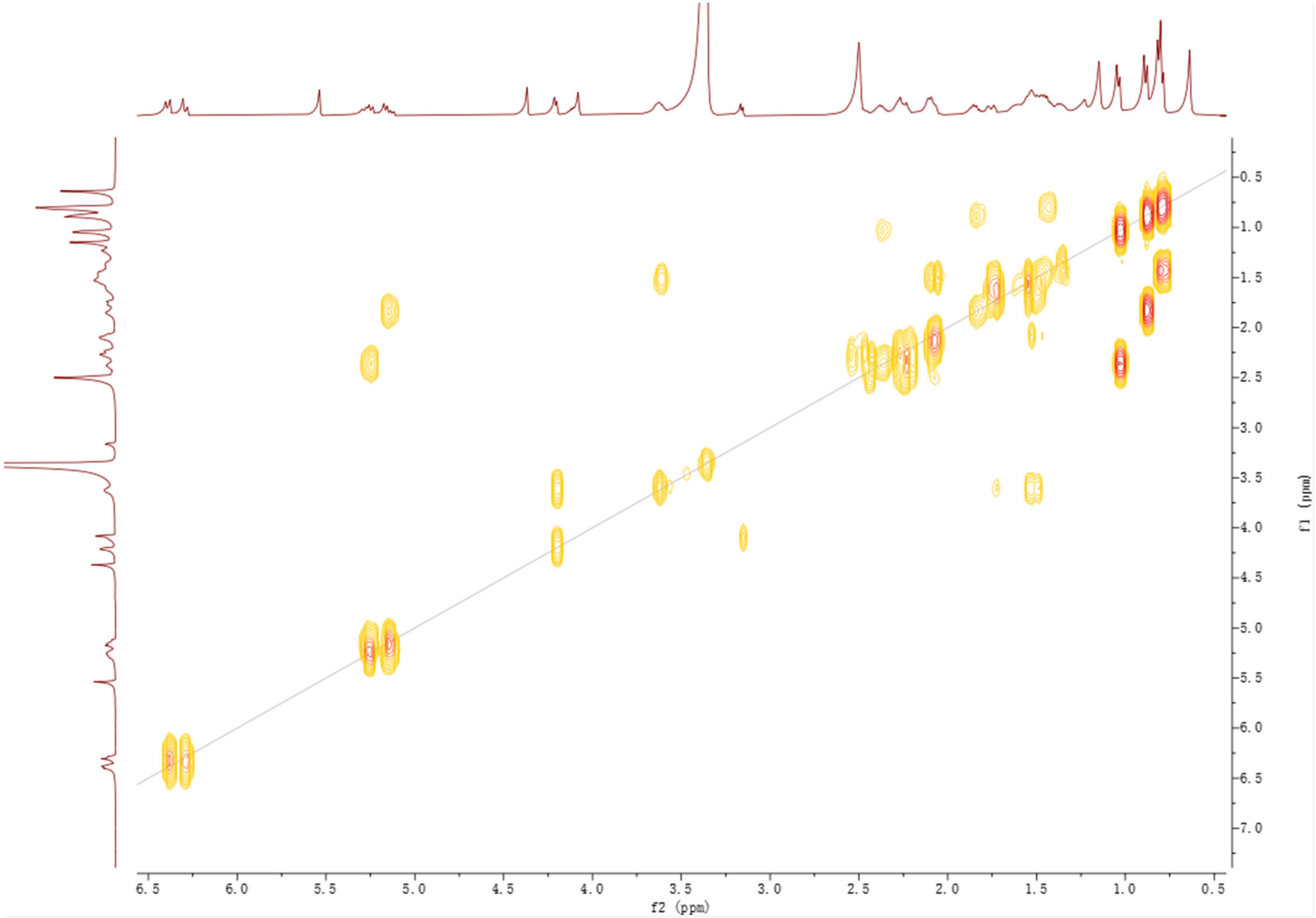

Figure S20. HMBC spectrum of compound **3**.

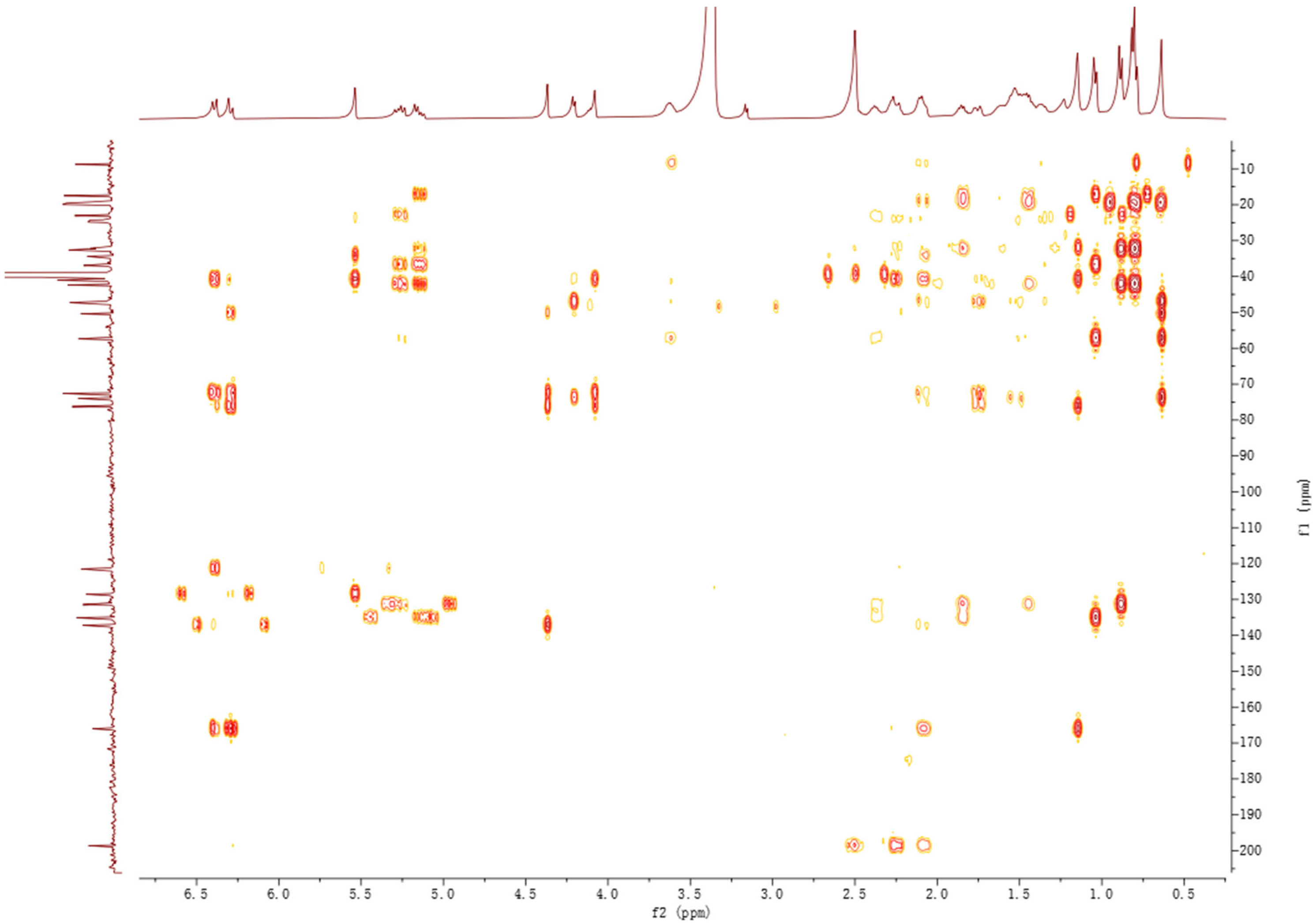

Figure S21. NOESY spectrum of compound **3**.

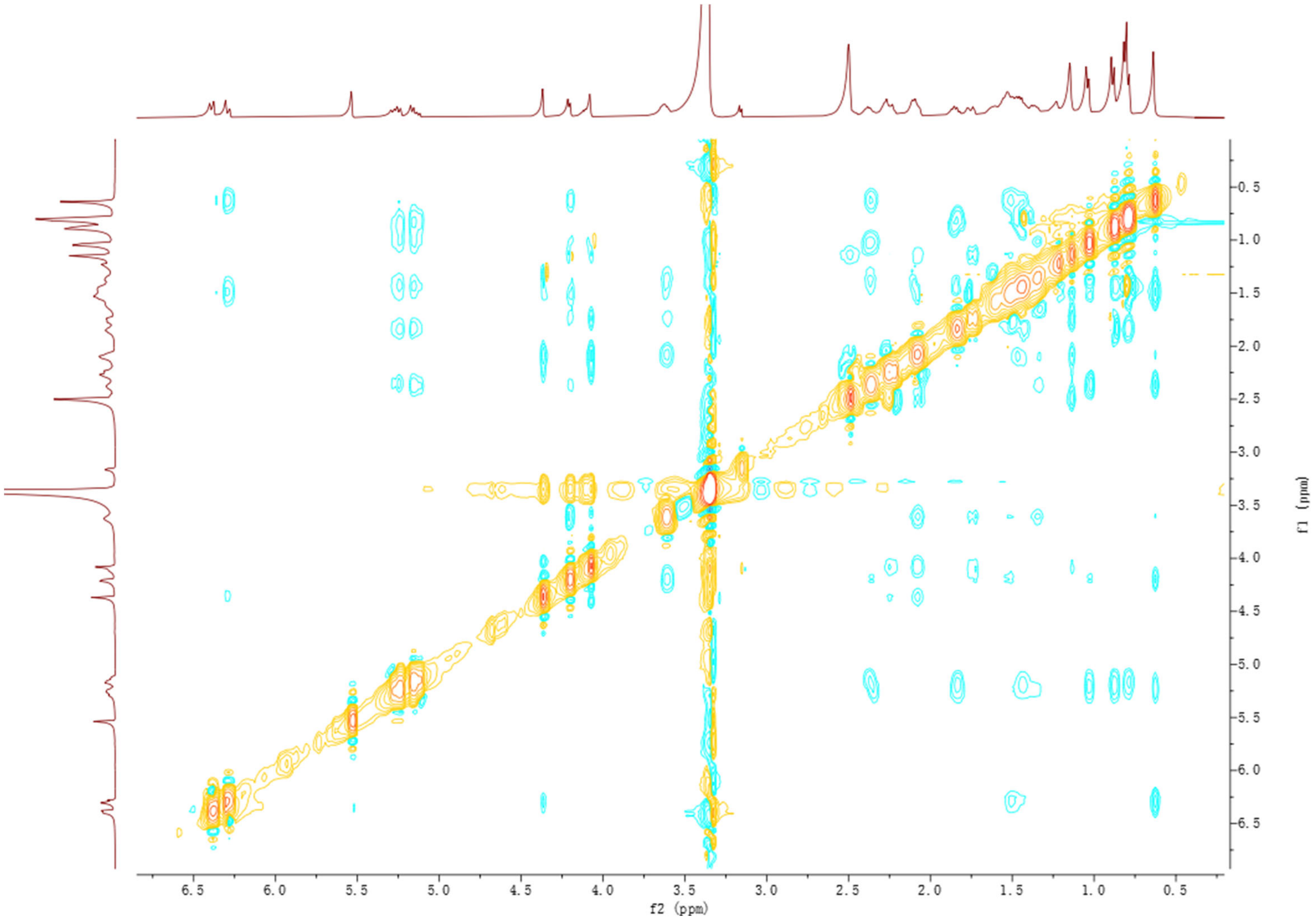

Figure S22. HRESI mass spectrum of compound **4**.

Elemental Composition Report

Page 1

Single Mass Analysis

Tolerance = 50.0 mDa / DBE: min = -1.5, max = 50.0
Element prediction: Off
Number of isotope peaks used for i-FIT = 3

Monoisotopic Mass, Even Electron Ions
26 formula(e) evaluated with 7 results within limits (up to 50 closest results for each mass)
Elements Used:
C: 0-40 H: 0-50 O: 2-7
ZQB-26-RE 1 (0.032)
1: TOF MS ES+

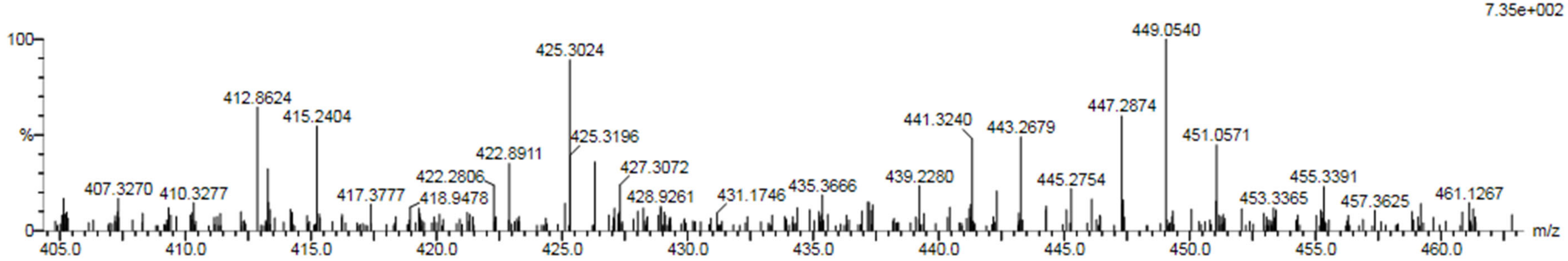

		mDa	PPM	DBE				
Minimum:				-1.5				
Maximum:		50.0	10.0	50.0				

Mass	Calc. Mass	mDa	PPM	DBE	i-FIT	Norm	Conf(%)	Formula
425.3024	425.3056	-3.2	-7.5	8.5	116.2	1.609	20.01	C28 H41 O3
	425.2903	12.1	28.5	4.5	116.4	1.784	16.79	C24 H41 O6
	425.3267	-24.3	-57.1	3.5	116.5	1.874	15.34	C25 H45 O5
	425.2692	33.2	78.1	9.5	116.5	1.920	14.66	C27 H37 O4
	425.3420	-39.6	-93.1	7.5	116.5	1.923	14.62	C29 H45 O2
	425.3478	-45.4	-106.7	-1.5	117.0	2.373	9.32	C22 H49 O7
	425.2539	48.5	114.0	5.5	117.0	2.379	9.26	C23 H37 O7

Figure S23. ^{1}H NMR (600 MHz, DMSO-d_6) spectrum of compound **4**.

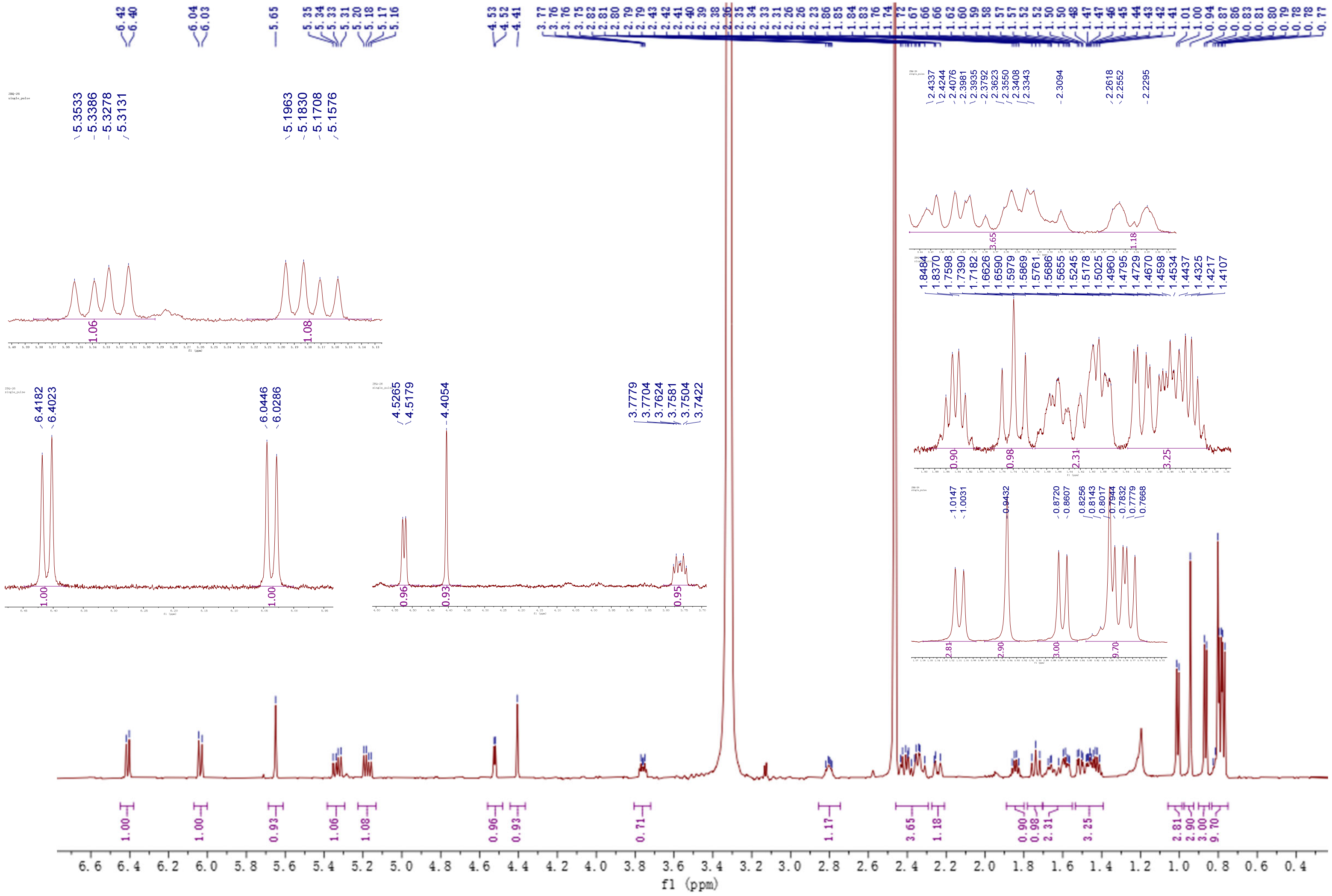

Figure S24. ^{13}C NMR (150 MHz, DMSO-d_6) spectrum of compound **4**.

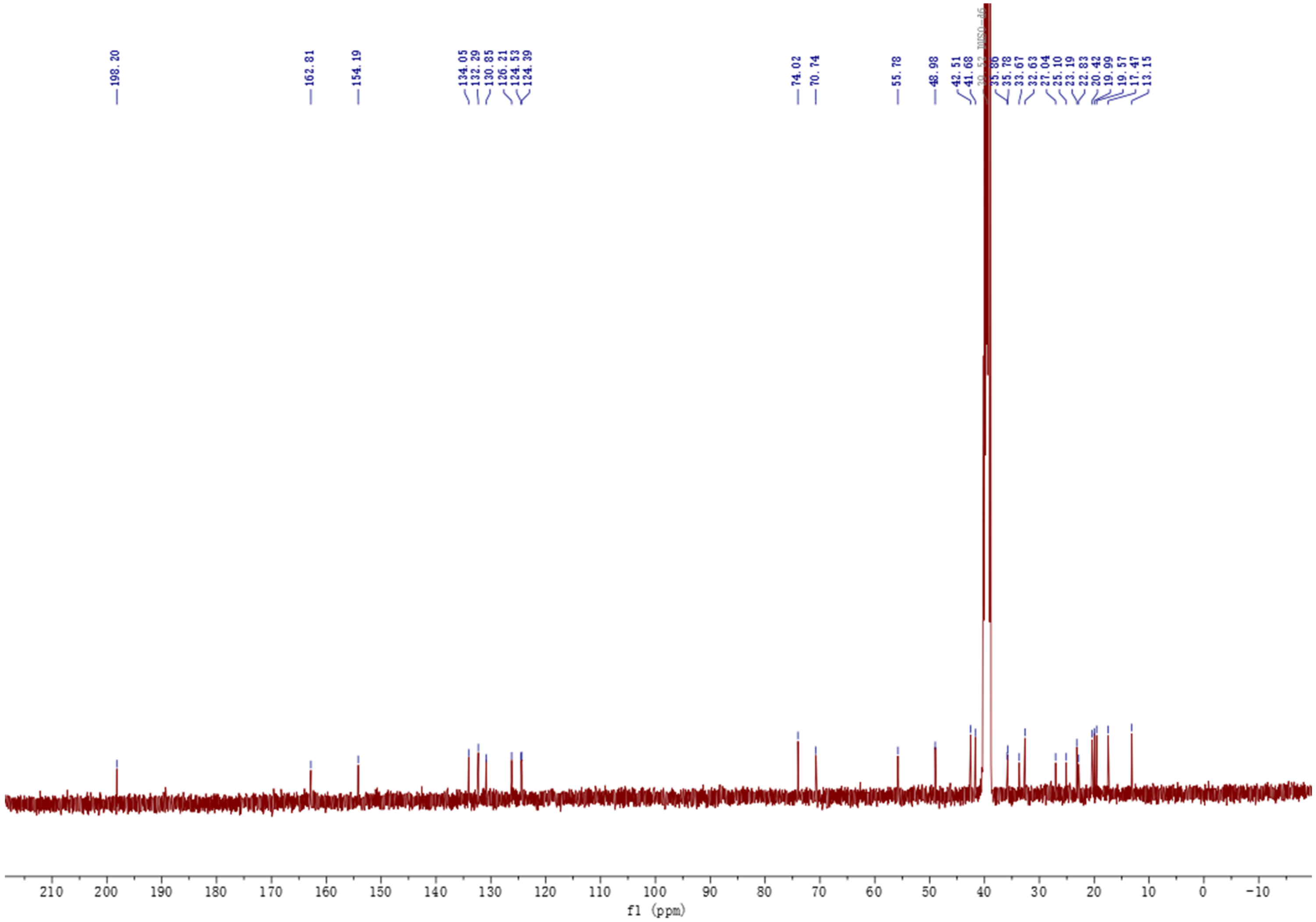

Figure S25. HSQC spectrum of compound **4**.

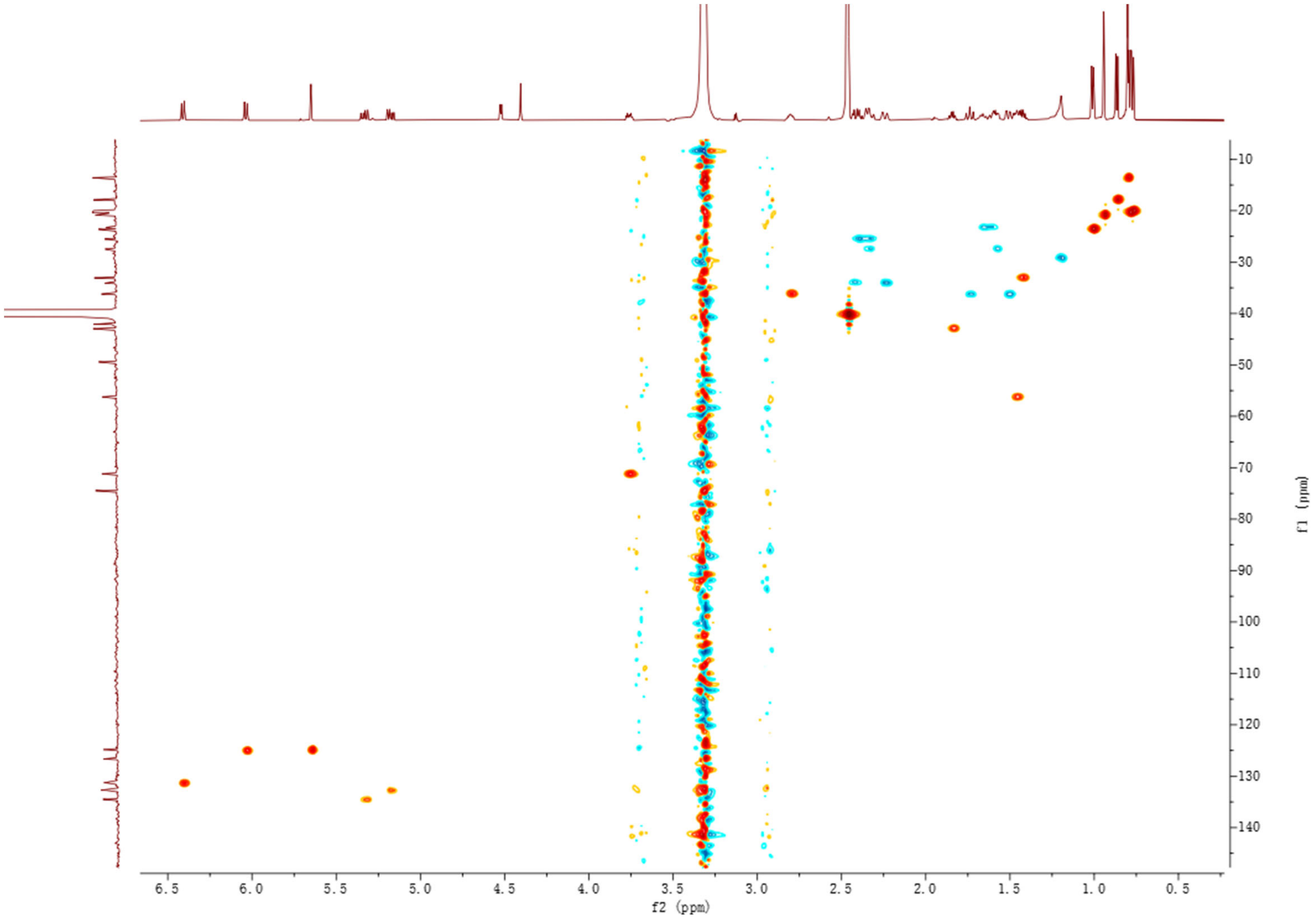

Figure S26. COSY spectrum of compound **4**.

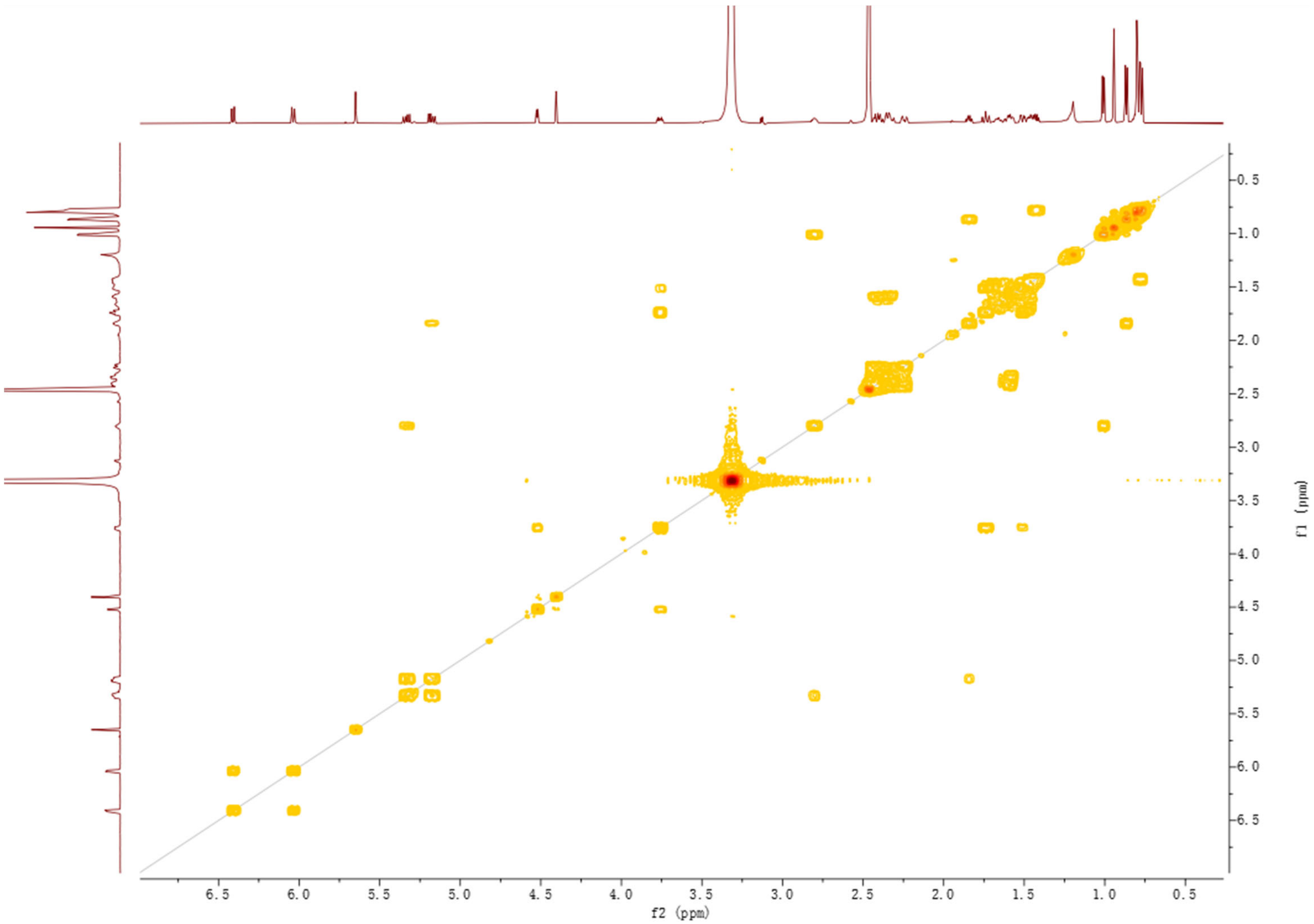

Figure S27. HMBC spectrum of compound **4**.

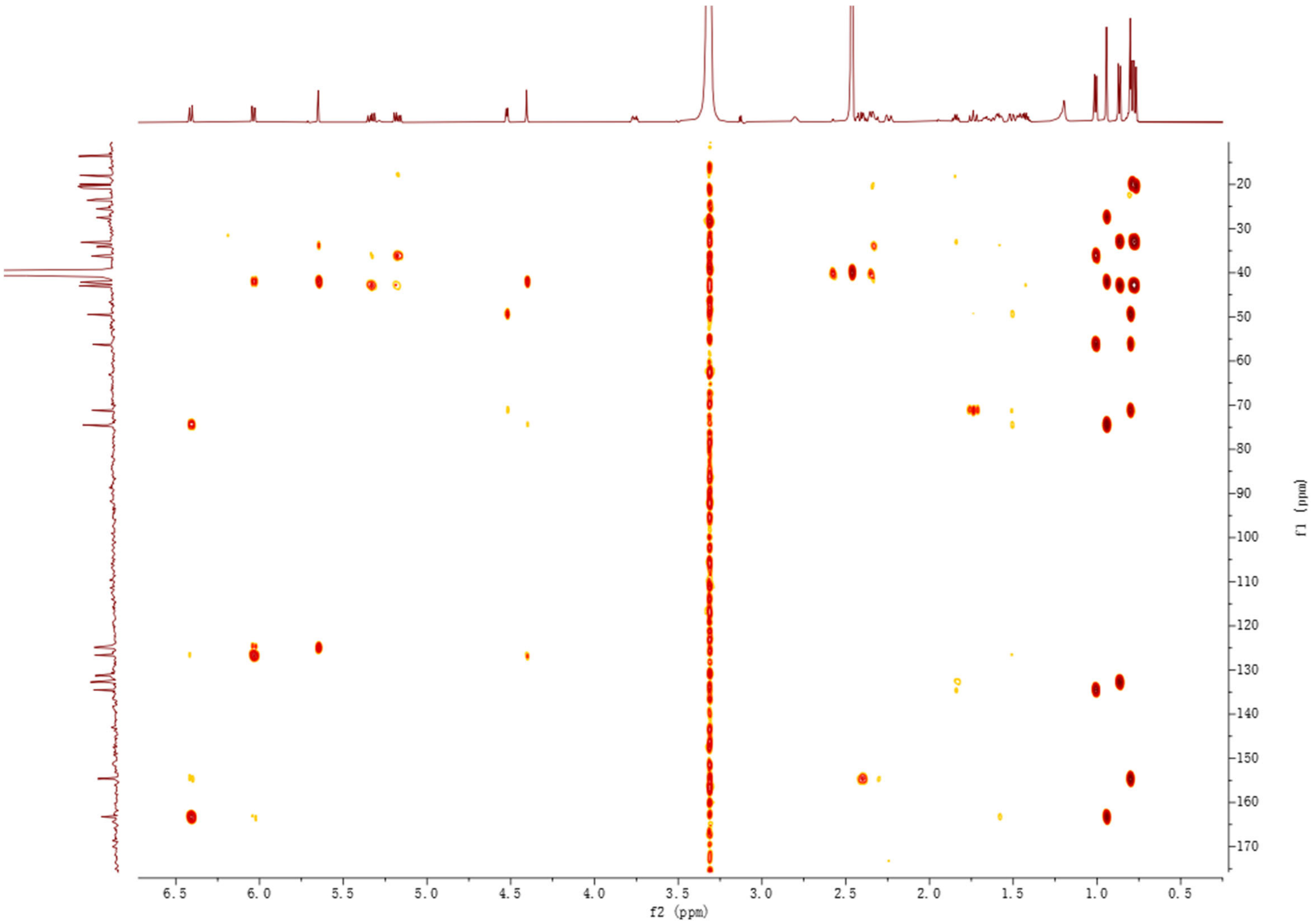

Figure S28. NOESY spectrum of compound **4**.

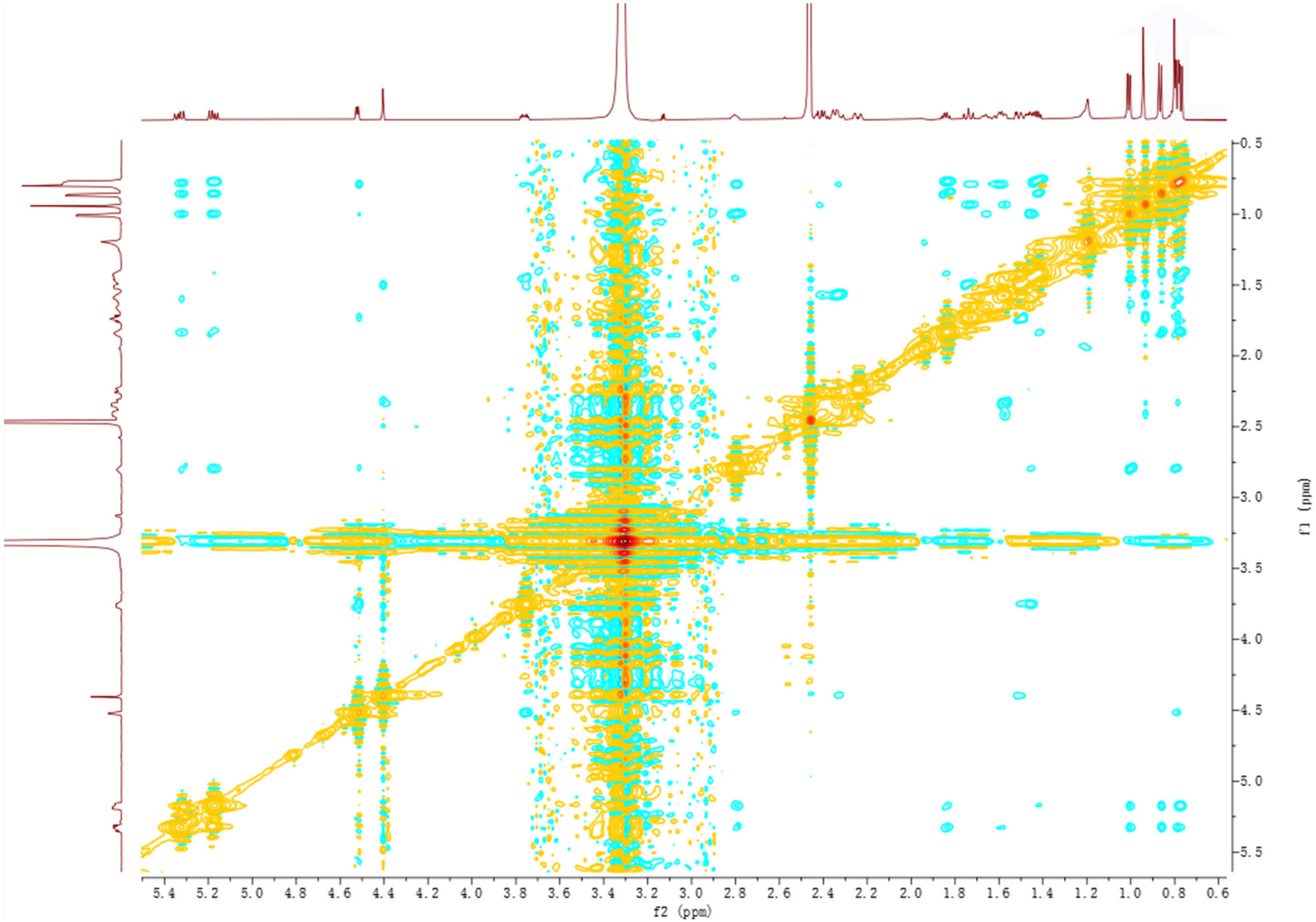

Figure S29. HRESI mass spectrum of compound **5**.

Elemental Composition Report

Single Mass Analysis

Tolerance = 50.0 mDa / DBE: min = -1.5, max = 50.0
Element prediction: Off
Number of isotope peaks used for i-FIT = 3

Monoisotopic Mass, Even Electron Ions
26 formula(e) evaluated with 6 results within limits (up to 50 closest results for each mass)
Elements Used:
C: 0-40 H: 0-50 O: 2-7
ZQB-25 1 (0.032)
1: TOF MS ES+

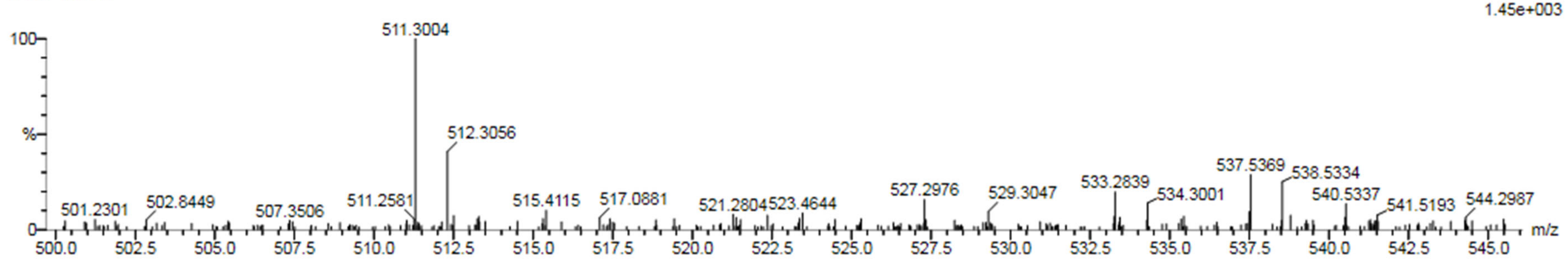

Minimum:				-1.5				
Maximum:		50.0	10.0	50.0				

Mass	Calc. Mass	mDa	PPM	DBE	i-FIT	Norm	Conf(%)	Formula
511.3004	511.3060	-5.6	-11.0	10.5	152.2	1.043	35.24	C31 H43 O6
	511.2848	15.6	30.5	15.5	152.6	1.435	23.82	C34 H39 O4
	511.3212	-20.8	-40.7	14.5	153.0	1.816	16.26	C35 H43 O3
	511.2696	30.8	60.2	11.5	153.3	2.122	11.98	C30 H39 O7
	511.2637	36.7	71.8	20.5	154.1	2.885	5.59	C37 H35 O2
	511.3423	-41.9	-81.9	9.5	153.8	2.644	7.11	C32 H47 O5

Figure S30. ^{1}H NMR (400 MHz, DMSO-d_6) spectrum of compound **5**.

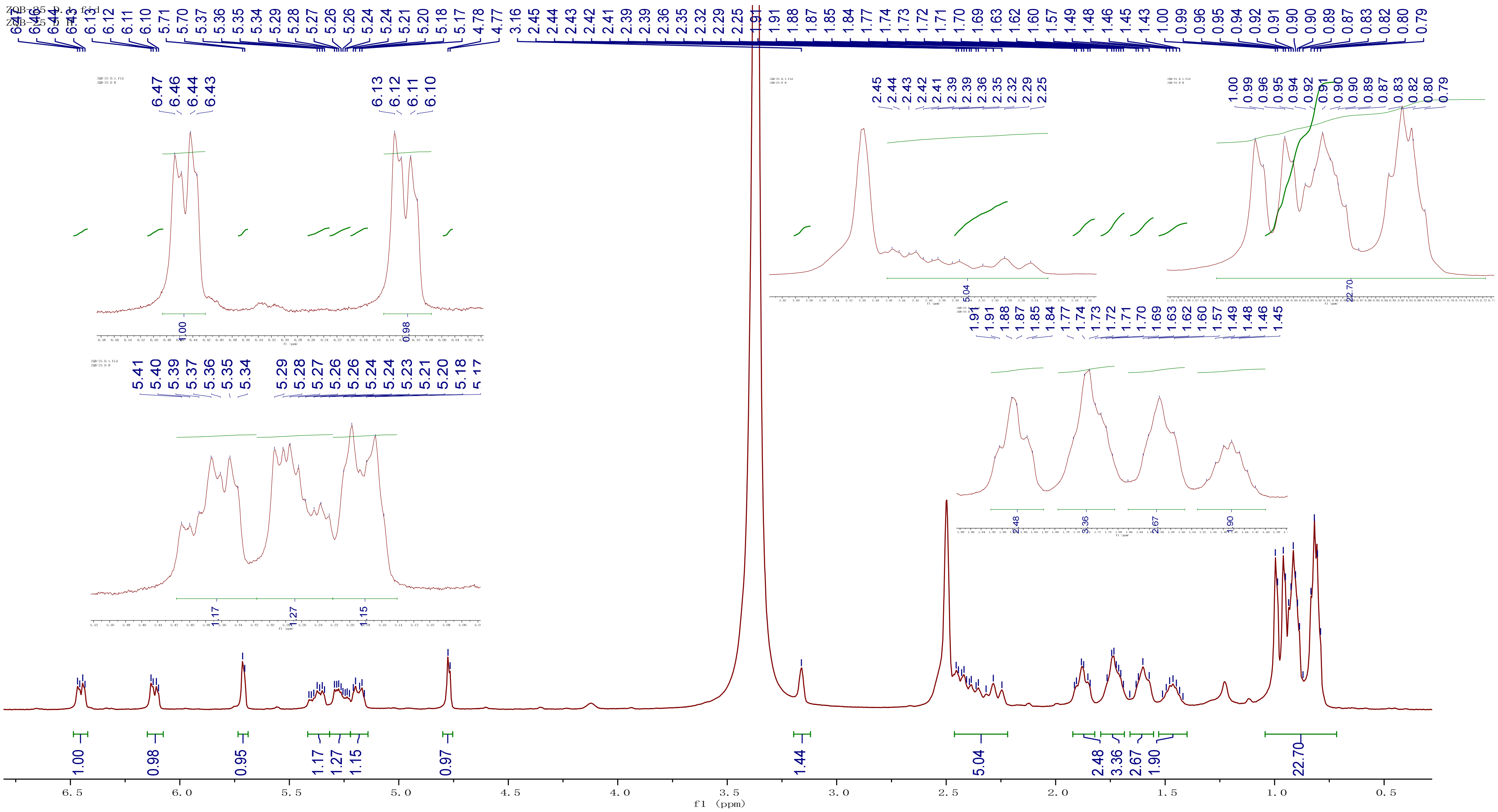

Figure S31. ^{13}C NMR (100 MHz, DMSO-d_6) spectrum of compound **5**.

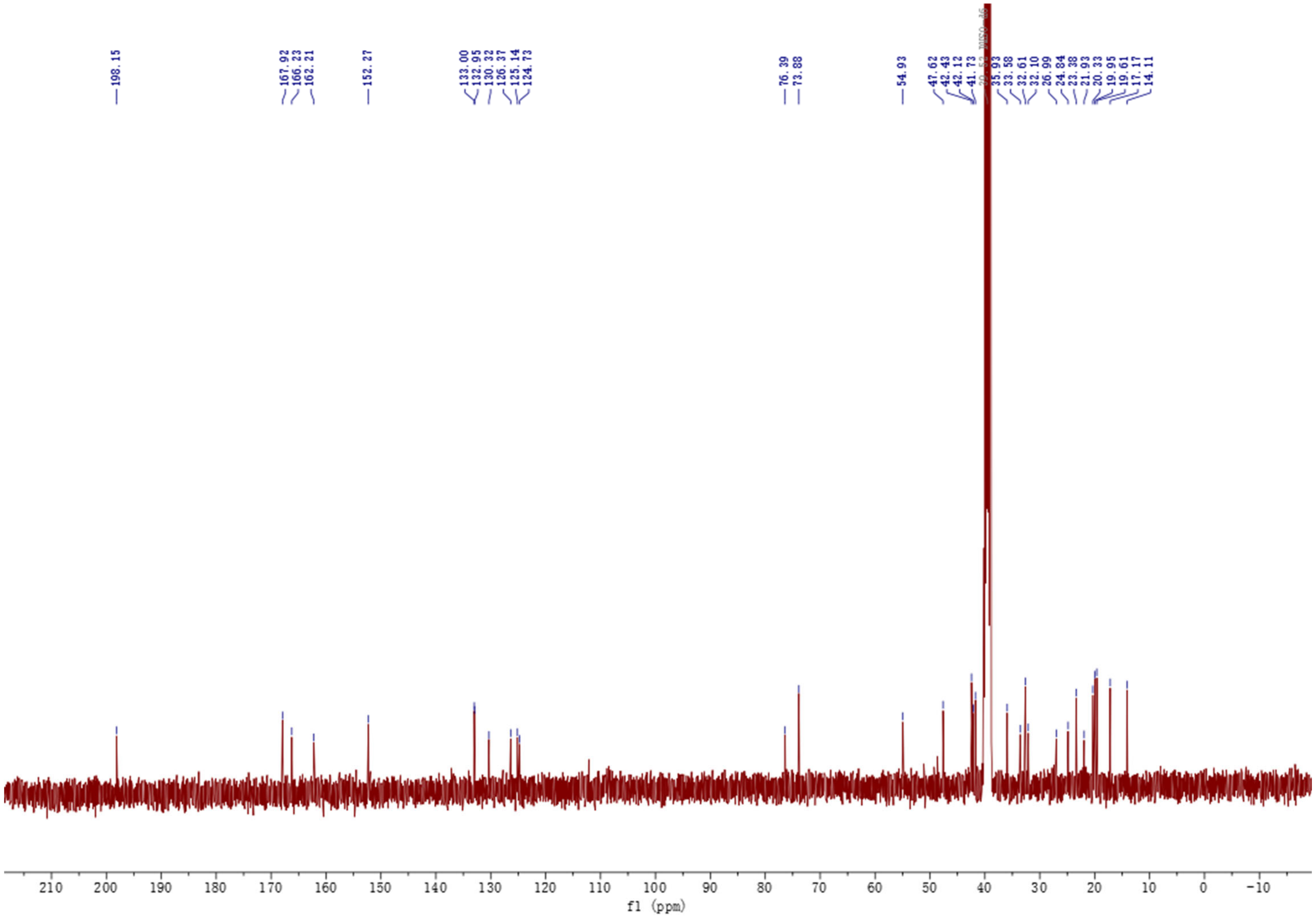

Figure S32. HSQC spectrum of compound **5**.

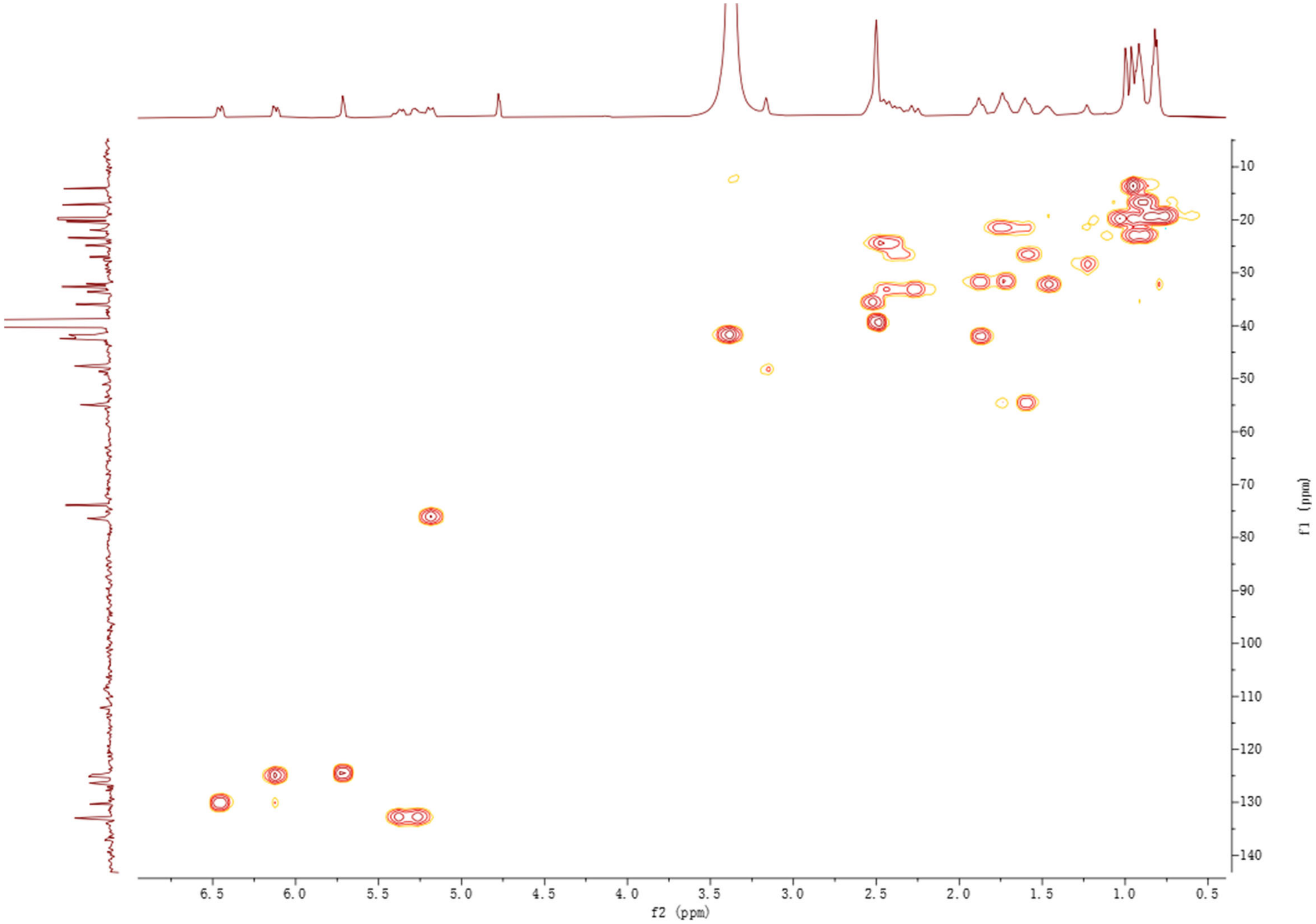

Figure S33. COSY spectrum of compound **5**.

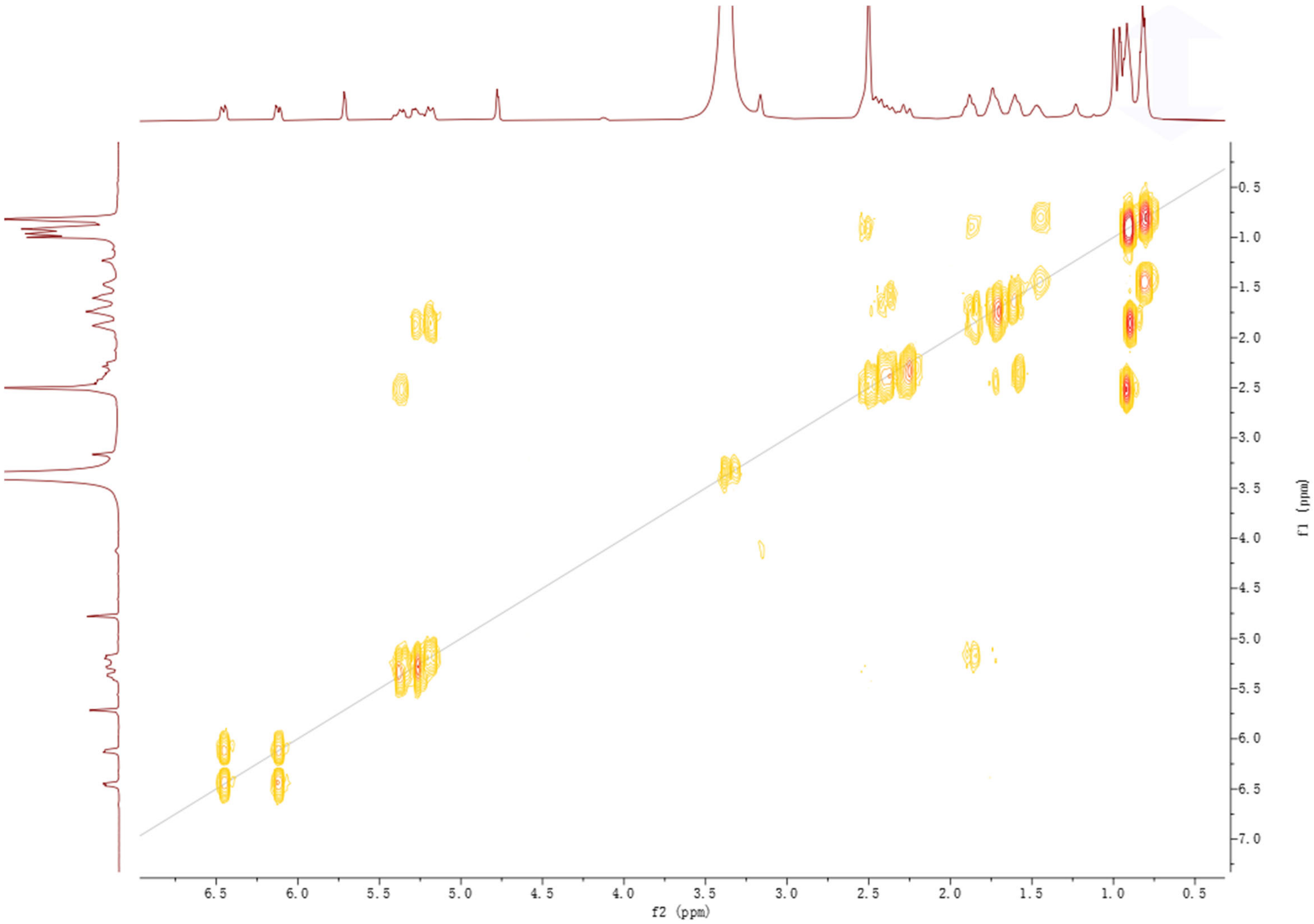

Figure S34. HMBC spectrum of compound **5**.

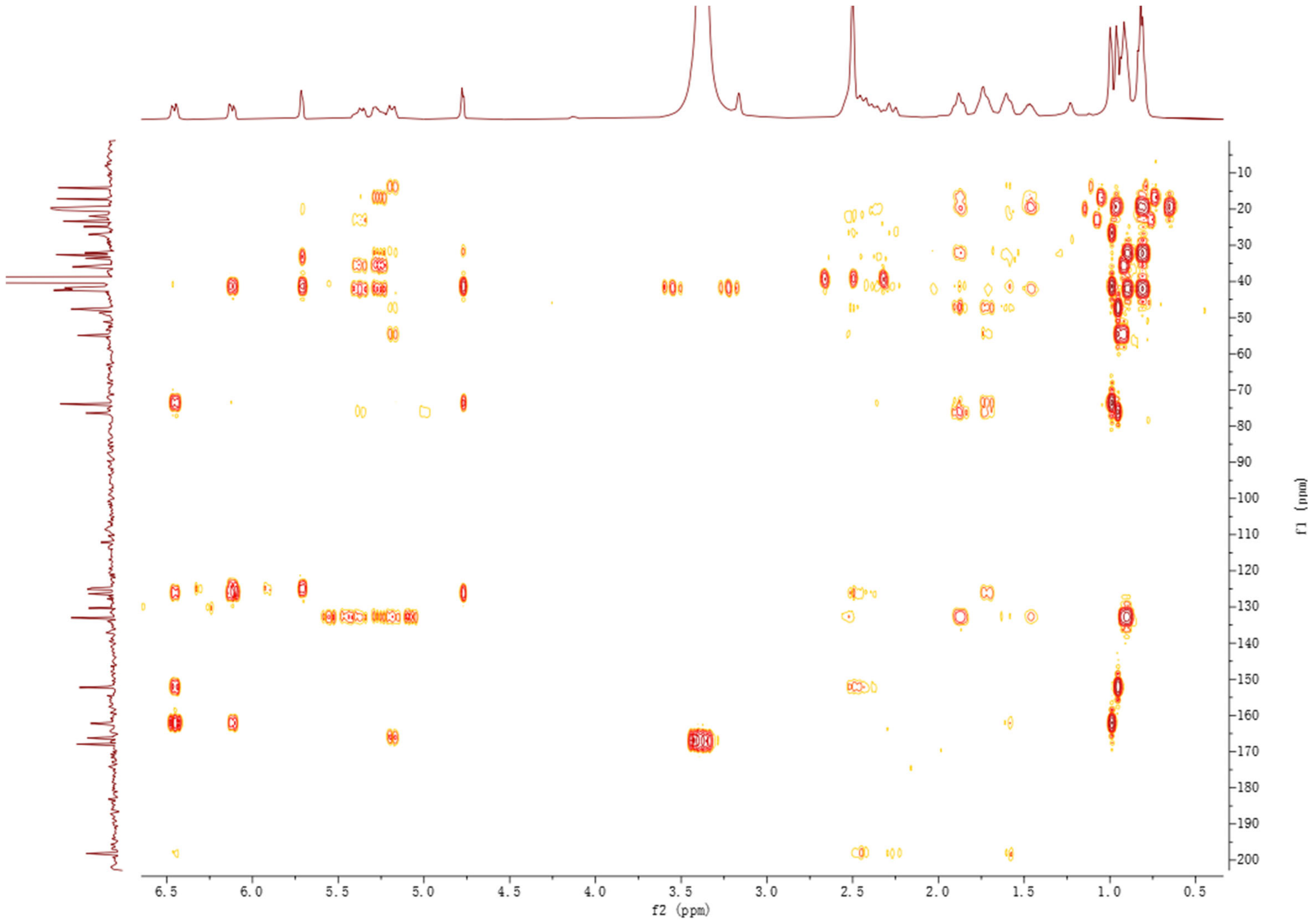

Figure S35. NOESY spectrum of compound **5**.

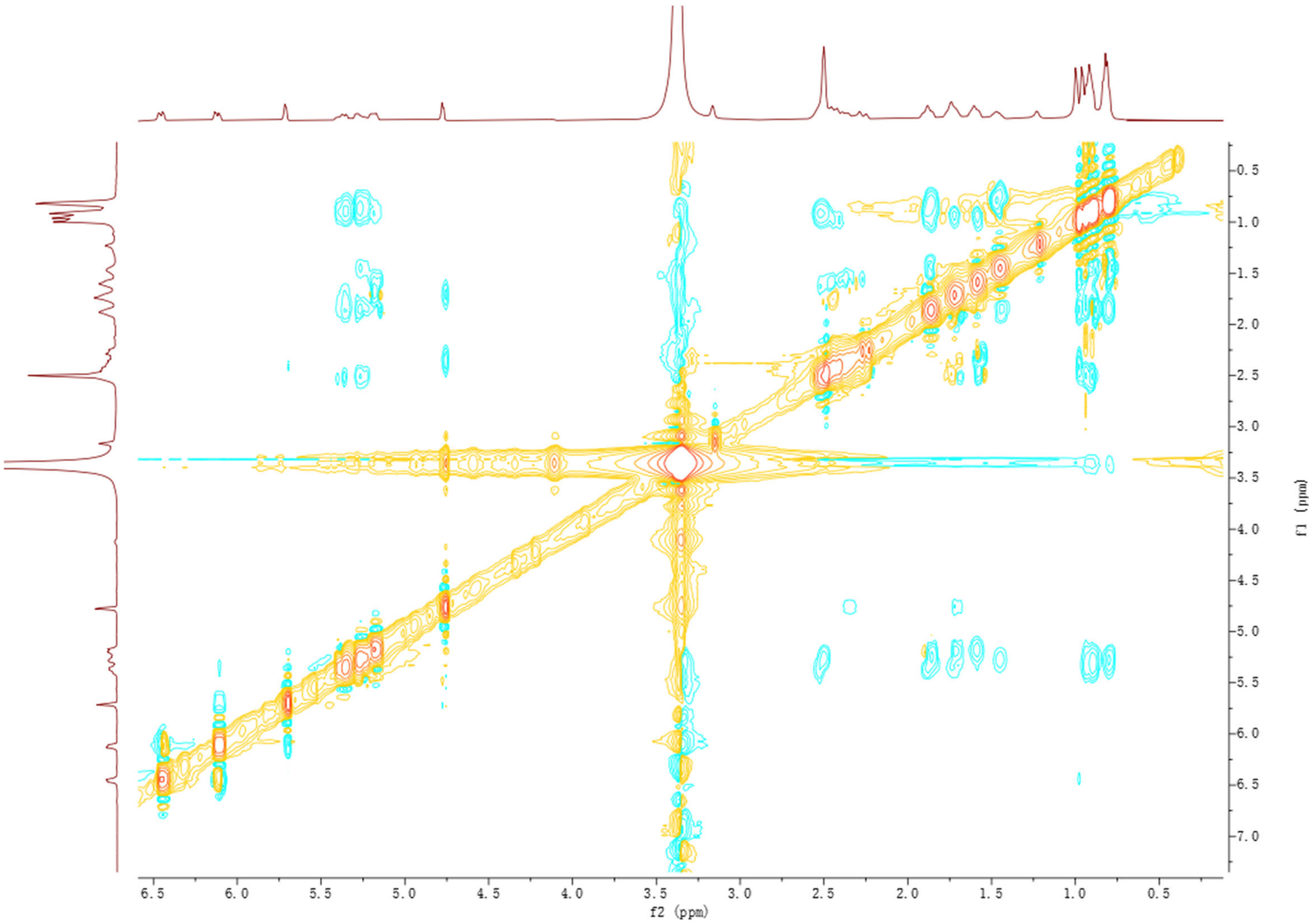

Figure S36. Cytotoxicity of chrysogenones A–E on unstimulated NRK-49F fibroblasts: Cell viability was assessed using a CCK-8 assay following a 24-hour treatment period. (A) NRK-49F cells were treated with 20 µM of compounds **1**–**5**. (B) Cells were treated with the indicated concentrations of compound **2**. Data are presented as mean ± SEM. Statistical significance was determined using one-way ANOVA followed by Bonferroni's multiple comparisons test. *$p < 0.05$ vs. ctrl group

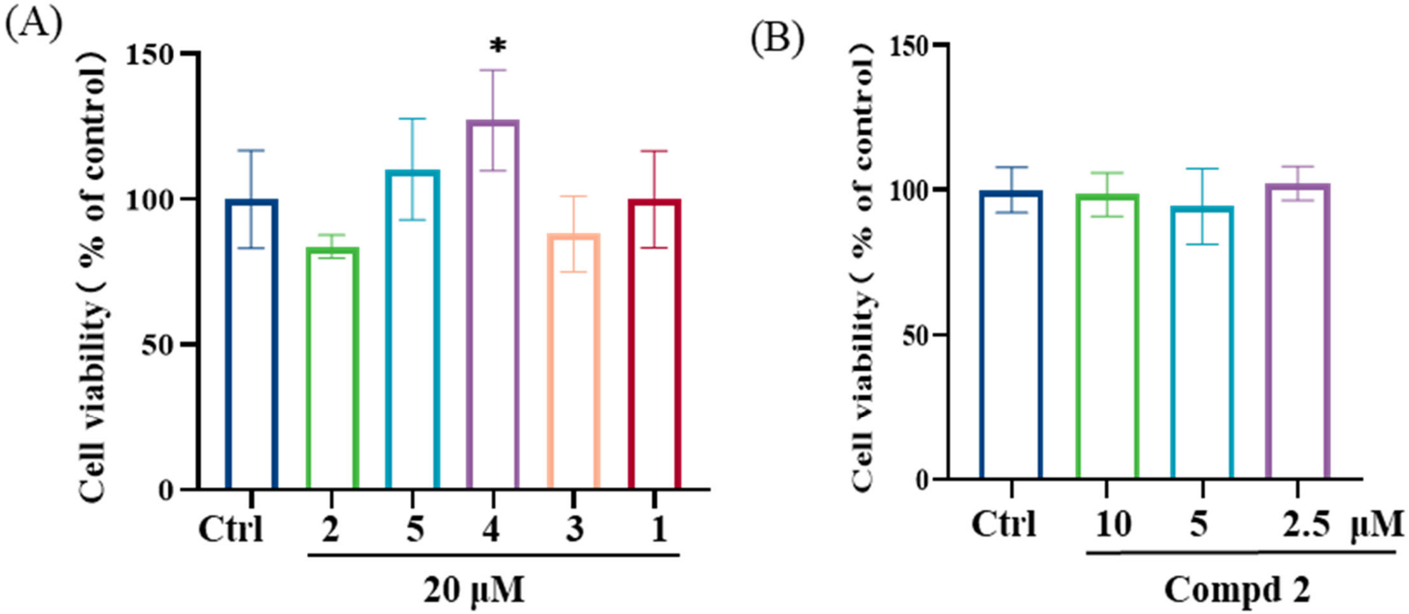

Supplement: Supplementary file 1 [file marinedrugs-24-00121-s001.zip › marinedrugs-4192102-supplementary.pdf]
